# Impact of an Alert-Based Inpatient Clinical Decision Support Tool to Prevent Drug-Induced Long QT Syndrome: Large-Scale, System-Wide Observational Study

**DOI:** 10.2196/68256

**Published:** 2025-04-14

**Authors:** Katy E Trinkley, Steven T Simon, Michael A Rosenberg

**Affiliations:** 1 Adult and Child Center for Outcomes Research and Delivery Science Center University of Colorado Anschutz Medical Campus Aurora, CO United States; 2 Division of Cardiology University of Colorado Anschutz Medical Campus Aurora, CO United States

**Keywords:** drug-induced QT prolongation, predictive modeling, electronic health records, clinical decision support, alert-based CDS system, tools, long QT syndrome, prevention

## Abstract

**Background:**

Prevention of drug-induced QT prolongation (diLQTS) has been the focus of many system-wide clinical decision support (CDS) tools, which can be directly embedded within the framework of the electronic health record system and triggered to alert in high-risk patients when a known QT-prolonging medication is ordered. Justification for these CDS systems typically lies in the ability to accurately predict which patients are at high risk; however, it is not always evident that identification of risk alone is sufficient for appropriate CDS implementation.

**Objective:**

In this investigation, we examined the impact of a system-wide, alert-based, inpatient CDS tool designed to prevent diLQTS across 10 known QT-prolonging medications.

**Methods:**

We compared the risk of diLQTS, duration of hospitalization, and in- and out-of-hospital mortality before and after implementation of the CDS system in 178,097 hospitalizations among 102,847 patients. We also compared outcomes between those in whom an alert fired and those in whom it did not, and within the various responses to the alert by providers. Analyses were adjusted for age, sex, race and ethnicity, inpatient location, electrolyte values, and comorbidities, with the latter processed using an unsupervised clustering analysis applied to the top 500 most common medications and diagnosis codes, respectively.

**Results:**

We found that the simple, rule-based logic of the CDS (any prior electrocardiograph with heart rate–corrected QT interval (QTc)≥500 ms) successfully identified patients at high risk of diLQTS with an odds ratio of 2.28 (95% CI 2.10-2.47, *P*<.001) among those in whom it fired. However, we did not identify any impact on the risk of diLQTS based on provider responses or on the risk of inpatient, 3-month, 6-month, or 1-year mortality. When compared with rates prior to implementation, the risk of diLQTS was not significantly different after the CDS tools were deployed across the system, although mortality was significantly higher after the tools were implemented.

**Conclusions:**

We found that despite successful identification of high-risk patients for diLQTS, deployment of an alert-based CDS did not impact the risk of diLQTS. These findings suggest that quantification of high risk may be insufficient rationale for implementation of a CDS system and that hospital systems should consider evaluation of the system in its entirety prior to adoption to improve clinical outcomes.

## Introduction

The expansion of electronic health record (EHR) systems has brought opportunities to apply automated clinical decision support (CDS) tools to guide day-to-day management decisions. These systems are attractive in that they can be scaled across an entire health system to provide guidance in the management decisions for potentially thousands of patients. When combined with the ability to directly import patient information from the EHR and apply algorithms ranging in complexity from simple rule-based systems to artificial intelligence, these tools have great potential to improve patient safety and clinical efficacy.

One of the conceptual models that is used as a rationale for selecting a clinical decision to target for CDS has been the idea of risk prediction, which is based on the idea that if one could alert the provider to the presence of an increased risk in an individual patient, actions could be taken to mitigate that risk. A number of CDS tools have been created based on this simple idea [1,2], and many more have been proposed as better risk-prediction models have been developed and published. In the past decades, much attention has been drawn to the importance of calibration and external validation of risk prediction models, with a specific focus on data shifts and transportability across populations [3,4], improving the rigor in which risk prediction itself is evaluated in considering the creation of a CDS system.

However, even if a risk-prediction model has been sufficiently vetted for accuracy and calibration, there remains the open question of what actions can be taken to mitigate risk and whether the system as a whole provides a meaningful impact on the desired outcome. While new medications or procedures are generally subject to randomized clinical trials to demonstrate effectiveness, such rigor is often overlooked in the design of CDS systems when the intervention is not as novel or discretely defined. These systems are often implemented based solely on the risk prediction itself, implying that if an accurate risk prediction model can indicate which patients are at high risk, then providers will “figure out” what to do with that information. They are rarely subject to formal randomized trials and, in many cases, are not even evaluated post deployment to assess effectiveness on the actual outcome.

An example of one such CDS system is that designed to prevent drug-induced QT prolongation (diLQTS) [5]. diLQTS, and the subsequent risk of torsade de pointes, is widely recognized as a clinical concern across health care systems due to the number of medications that can cause diLQTS, including many used for noncardiac indications [6]. A number of studies have identified clinical and genetic [7] risk factors for diLQTS, with risk-prediction models ranging from simple [5,8], rule-based algorithms (ie, any prior electrocardiograph [ECG] with heart rate–corrected QT interval (QTc) longer than 500 ms) to complex deep learning models [9,10]. Although few complex models have been implemented in CDS for diLQTS (including within our own health system), it has been suggested that better risk prediction could improve the effectiveness of CDS tools designed to prevent diLQTS.

The limitation of adequate risk prediction was one explanation for a prior finding by our group in the evaluation of a rule-based, alert-based CDS system, which found that mortality was paradoxically decreased in patients in whom the provider chose to ignore the recommendations of the CDS [11]. While these results were unexpected and notable, they were generally dismissed based on the limits of the dataset. It was not evident that the alert was indeed identifying patients at high risk of diLQTS, as we did not examine any subjects without an alert. The mortality signal was explained by the assumption that the action of the provider was an indicator of sicker patients, although this explanation could not be directly evaluated due to limited information about comorbidities or location (ie, intensive care unit [ICU] versus floor) of each patient. In addition, that study did not perform any evaluation of the CDS itself in terms of impact, as all subjects included had the alert fire; there was no control group available for comparison.

In this follow-up investigation, we applied data extraction techniques to obtain patient-specific comorbidities, including medications, diagnoses, location, and laboratory measures, to evaluate the impact of the CDS to prevent diLQTS. In addition to the collection of data about those in whom the alert fired, we also collected data about those who were prescribed a known QT-prolonging medication without having the alert fire, including those treated prior to the deployment of the CDS and those not identified by the rule-based system as being at high risk. The goal of this investigation is to examine the hypothesis that the CDS alert system reduced the risk of diLQTS and explore patient characteristics, clinic settings, and provider responses to the alert that could have impacted diLQTS as well as hospitalizations and mortality.

## Methods

### Data Source and Study Population

The population has been described in detail previously [9-11]. Briefly, we extracted EHR data for any inpatient aged 18-90 years, treated in a UCHealth facility for any indication, in whom a known QT-prolonging medication was administered that contained a corresponding CDS that deploys in an interrupted manner electronically. The list of culprit medications includes ondansetron, haloperidol, azithromycin, levofloxacin, escitalopram, citalopram, sotalol, droperidol, methadone, and hydroxychloroquine. The index visit was defined as the first order of a culprit medication for a given patient on a given hospitalization (encounter), with only one medication used to define each hospitalization for each patient. As such, a given individual patient could be included more than once, although each encounter (hospitalization) was only used once for each patient under a given culprit medication. The earliest date of enrollment was July 6, 2011, and the latest was April 16, 2024.

### CDS Alert System

The CDS alert system uses a native, rule-based logic system within Epic called the BestPractice Advisory (BPA). To avoid confusion in terminology, we refer to the “BPA” as the specific alert that fires for a given patient with a given culprit medication; we refer to “CDS” as the system as a whole, including those in whom the BPA did not fire due to not meeting criteria. The logic of the CDS is the electronic order for a culprit medication in a patient in whom a prior ECG had a recorded QTc of ≥500 milliseconds (rate-corrected QTc based on Bazett’s formula). The alert is interruptive, firing at the time of medication order entry and requiring the provider to take a given action to proceed within the system. The specific *provider action* records how the provider responded to the alert within the BPA prompt and included the following categories: Cancel BPA, Acknowledge or Override warning, Accept BPA (no action taken), and Remove single order. The latter is considered alert compliance, and the former 3 were considered noncompliance or ignore. These 2 categories constituted the *action group* of the alert. Only the first BPA alert for a given medication encounter was used. The specific implementation dates by culprit medication include sotalol June 2015; methadone July 2016; citalopram July 2016; droperidol July 2016; haloperidol November 2011; ondansetron July 2016; azithromycin July 2016; escitalopram July 2016; levofloxacin September 2016; and hydroxychloroquine March 2020.

### Description of Extraction Process

For a detailed description of the data extraction process, see Methods in Multimedia Appendix 1. In brief, data was collected retrospectively, with extraction conducted using the Health Data Compass EHR repository, an institutional resource containing a full copy of the EHR backend for research and analysis. The database was queried to identify those encounters (ie, hospitalizations) where the BPA fired, as well as those in whom one of the culprit QT-prolonging medications was ordered electronically. The latter group includes those who were treated prior to the deployment of the drug-specific CDS, as well as those who were treated after but were not identified as being high risk (ie, QTc≥500 ms). The first order for each culprit medication was used, such that an individual patient could have multiple entries if multiple culprit medications were prescribed on a given encounter, but only one entry per culprit medication per encounter. Leveraging the longitudinal capacity of the EHR for time-dependent events, we obtained timestamps for all data elements extracted, from which were calculated time intervals linked to the start of the encounter (ie, time and date of admission). Outpatient mortality was adjudicated as being within 3 months, 6 months, and 1 year of the index encounter beginning. The final dataset included fully deidentified data, and no protected health information was included. Access to the dataset is restricted to investigators covered under the Colorado Multiple Institution Internal Review Board; to request access, contact the corresponding author or institutional representative.

### ECG Data Analysis

ECG data were extracted for all ECGs within the system (across all encounters) for each subject in the dataset in which a QT interval was present in electronic format. The QRS and QTc was calculated as QTc=QT interval–max (0, QRS interval–100)/(RR interval)^1/3^. For QTc measurements prior to the medication order, the maximum and mean QTc were recorded for each encounter (maxPreQT and meanPreQT, respectively). We then obtained all ECGs performed within 1 hour after the medication order or BPA alert (whichever was later) and calculated the maximum and mean QTc (maxPostQT and meanPostQT, respectively). The diagnosis of diLQTS was defined as any postmed ECG with maximum QTc≥500 milliseconds or a change in maximum QTc over 60 milliseconds (post- minus pre-).

### Clinical Factors

For each encounter, the age, sex, race, and ethnicity were recorded based on the time of the BPA alert or medication order (which was earliest). Using these same criteria, we also extracted all diagnosis codes (*ICD-9* [*International Classification of Diseases, Ninth Revision*] or *ICD-10* [*International Statistical Classification of Diseases, Tenth Revision*]) present on the problem list and all medications in the inpatient medication list that were ordered prior to the BPA alert or medication order (whichever was earliest). Laboratory values for potassium, magnesium, and creatinine were recorded based on the closest time to the BPA alert or medication order, but no longer than 24 hours before or after. The patient location was recorded according to when the BPA alert fired or medication was ordered, which were grouped into the following categories: ICU, telemetry, obstetrics/gynecology floor (OB/Gyn), and stepdown. ICU and stepdown were mutually exclusive, but telemetry and OB/Gyn could include patients in the ICU or stepdown as well (all ICU patients were also assigned to telemetry).

### Creation of Patient Clusters

In prior work, we examined the use of patient clusters to define risk groups, in which we identified a cluster number of 4 to be optimal [9]. To repeat this process, we first categorized age into bins of under 40, 40-60, 60-80, and 80 years or older. Diagnoses and medications were then one-hot encoded into individual diagnoses and medications (present yes or no), and the top 500 most common medications and top 500 most common diagnoses were kept for cluster analysis. The 500th most common diagnosis was insomnia (n=554, 0.31%), and the 500th most common medication was cholecalciferol (n=1585, 0.89%), suggesting that clustering larger numbers of either was unlikely to have a significant impact on cluster assignment. Encounters were then clustered based on age category, sex, race, ethnicity, inpatient location, and the 500 most common medications and diagnoses. To identify the optimal cluster number, K-means clustering with Hamming distance was applied for k=2-30 clusters on a sample of 10,000 subjects, from which the inertia scores were calculated. Using the elbow method, we confirmed that a cluster number of 4 provided reasonable separation of patients (Figure S1A in Multimedia Appendix 1), which was also validated using principal component analysis (Figure S1B in Multimedia Appendix 1). We then calculated the cluster number for the entire set of encounters among 4 groups.

### Analysis

The primary outcome of the analysis was the presence of diLQTS, with secondary outcomes of hospital duration and inpatient, 3-month, 6-month, and 1-year mortality. For binary outcomes, marginal logistic regression models were developed; for hospital duration, marginal Poisson regression models were used. Marginal models were fit using generalized estimating equations with exchangeable correlation within individual patients. Unless otherwise specified, models were adjusted for age, sex, race and ethnicity, location, cluster, pretreatment QTc, and closest magnesium, potassium, and creatinine values (also adjusted for timing related to medication administration), with interaction terms for the culprit medication, alert firing, and actual administration, where applicable, with a goal to examine the impact of the alert and the medication on risk among those in whom the medication was administered. Note that in several instances, a medication was ordered or administered separate from the intended alert system. To account for these discrepancies, these situations were recoded as missing. Specifically, the 441 encounters in which the order was placed over 48 hours after the alert, the 346 encounters in which the medication was administered 72 hours after the alert, the 1846 encounters where the order was signed without a record of medication administration, and the 355 encounters in which the medication was administered without an order were all recoded as missing, and thus excluded from the analysis. For basic comparisons of clusters, categorical variables were compared using Chi-square test, and continuous variables were compared using one-way ANOVA. Stata SE (version 18) was used for statistical models. Scikit-learn (version 1.1.2; StataCorp) and Python (version 3.9.7) were used for K-means clustering, inertia scores, and principal component analysis.

### Ethics Approval

The protocol was approved by the University of Colorado Internal Review Board (COMIRB # 24-0200). Informed consent was waived due to the use of de-identified data in this retrospective, observational study. No images or other identifiable information was used in this investigation. No generative artificial intelligence was used for any portion of this manuscript. 

## Results

The dataset included 102,847 patients with 178,097 hospitalizations (encounters). The range of the number of hospitalizations per patient varied from 1 (N=63,165 patients) to 52 (N=52 patients). Table 1 provides baseline characteristics by cluster, as well as for the whole population of encounters. All variables were significantly different across clusters. Table S1 in Multimedia Appendix 1 provides the most common diagnoses and medications in each cluster. Table S2 in Multimedia Appendix 1 provides a list of the most different diagnoses by cluster. Clusters 0 and 3 contained less severe diagnoses and were associated with lower mortality than clusters 1 and 2 (Table 1).

**Table 1:** Demographics and outcomes by cluster. Shown are the mean (SD) for continuous measures and the number and percentage for categorical variables (of the cluster for each, respectively, as well as the total population). For each cluster, below each measure is a population-standardized measure indicating deviation from the overall population. For continuous measures, these values are the standard mean difference (SMD=mean of cluster–population mean/population standard deviation). For categorical measures, these values are the odds ratio (OR), calculated as the ratio of odds within the cluster and for the overall population (note: odds=percentage/1–percentage). Statistical comparison across all variables had *P*<.001 (t test, continuous measures) or *P*<.001 (Chi-square test, categorical variables).

		Cluster					
		0	1	2	3	Total	
Participants, n		8609	46,258	12,981	110,249	178,097	
Age (years), mean (SD)		62.73 (16.24)	63.34 (16.57)	56.71 (16.69)	57.46 (18.42)	59.19 (17.94)	
	SMD	+0.20	+0.23	–0.14	–0.10	—^a^	
Female sex, n (%)		4484 (52.09)	23,102 (49.94)	5660 (43.60)	60,435 (54.82)	93,681 (52.60)	
	OR	0.98	0.90	0.70	1.09	—	
White race, n (%)		7071 (82.1)	36,419 (78.73)	9165 (70.60)	79,913 (72.48)	132,568 (74.44)	
	OR	1.58	1.27	0.82	0.90	—	
Hispanic ethnicity, n (%)		1068 (12.41)	5778 (12.49)	2255 (17.37)	17,660 (16.02)	26,761 (15.03)	
	OR	0.80	0.81	1.19	1.08	—	
ICU^b^ care, n (%)		1539 (17.88)	13,108 (28.34)	8744 (67.36)	11,620 (10.54)	35,011 (19.66)	
	OR	0.89	1.62	8.43	0.48	—	
Telemetry, n (%)		3570 (41.47)	25,923 (56.04)	10,599 (81.65)	30,712 (27.86)	70,804 (39.76)	
	OR	1.07	1.93	6.74	0.59	—	
Stepdown unit, n (%)		1533 (17.81)	11,802 (25.51)	5156 (39.72)	10,591 (9.61)	29,082 (16.33)	
	OR	1.11	1.76	3.38	0.54	—	
OB/Gyn^c^, n (%)		55 (0.64)	178 (0.38)	24 (0.18)	787 (0.71)	1044 (0.59)	
	OR	1.09	0.66	0.31	1.22	—	
Max preQTc^d^		422.07 (54.02)	422.92 (59.86)	423.56 (65.87)	421.55 (56.10)	422.07 (57.77)	
	SMD	0.00	+0.01	+0.03	–0.01	—	
Mean preQTc^e^		398.10 (40.79)	392.08 (43.56)	384.23 (46.00)	393.66 (41.10)	392.78 (42.20)	
	SMD	+0.13	–0.02	–0.20	+0.02	—	
diLQTS^f^, n (%)		666 (7.7)	3929 (8.5)	1621 (12.5)	8474 (7.7)	14,690 (8.25)	
	OR	0.93	1.03	1.59	0.93	—	
Alert fired, n (%)		534 (6.20)	4278 (9.25)	1884 (14.51)	8267 (7.50)	14,963 (8.40)	
	OR	0.72	1.11	1.85	0.88	—	
Inpatient mortality, n (%)		118 (0.42)	2503 (5.41)	1538 (11.85)	1592 (1.44)	5751 (3.23)	
	OR	0.42	1.71	4.03	0.44	—	
3-Month mortality, n (%)		368 (4.27)	4995 (10.80)	2292 (17.66)	4605 (4.18)	12,260 (6.88)	
	OR	0.60	1.64	2.90	0.59	—	
6-Month mortality, n (%)		493 (5.73)	5909 (12.77)	2582 (19.89)	5962 (5.41)	14,946 (8.39)	
	OR	0.66	1.60	2.71	0.62	—	
1-Year mortality, n (%)		651 (7.56)	7020 (15.18)	2917 (22.47)	7775 (7.05)	18,363 (10.31)	
	OR	0.71	1.56	2.52	0.66	—	

^a^Not applicable.

^b^ICU: intensive care unit.

^c^OB/Gyn: obstetrics/gynecology floor.

^d^Max preQTc: maximum QTc of all ECGs prior to drug order.

^e^Mean preQTc: mean QTc of all ECGs prior to drug order.

^f^diLQTS: drug-induced QT prolongation on any ECG after drug order.

### Response to Alerts for QT Prolongation

We then sought to assess the impact of providers’ responses to the BPA alert. The BPA fired in 14,963 (8.4%) of 178,097 total encounters at which a culprit medication was ordered, of which 8985 (60.13%) complied with the alert within the BPA (action group), 5957 (39.87%) selected an action indicating noncompliance, and 21 (0.14%) were missing a BPA response. We also assessed compliance with the CDS by examining whether the provider actually ordered the medication after the BPA fired and whether it was then administered. Of the 14,963 alerts, the culprit medication was ordered and signed (noncompliance) in 4816 (32.19%) encounters within 48 (mean 3.6, SD 8.7) hours of the alert. Of these 4816 encounters, in 3298, the culprit medication was administered within 72 (mean 8.6, SD 14.3) hours after the BPA alert.

Table 2 displays the association of patient characteristics with BPA alert firing, action compliance, order compliance, and administration compliance (after filtering out missing). The proportion of medication orders in which the alert fired increased with age and was more common in the ICU or telemetry floors, as well as with certain medications, such as sotalol (Table 2).

**Table 2:** Summary of responses to BPA^a^ for drug-induced QT prolongation. Note that the percentage of BPA fired refers to what percentage of subjects within that group had the BPA fire; the percentage of order and administration compliance refers to what percentage of BPAs that fired had the order placed or medication administered, respectively. The difference in totals reflects missingness due to discrepancies in orders placed 48 hours after the alert, administered 72 hours after the alert, ordered without administration, or administered without an electronic order.

		Participants, n	BPA fired, n (%)	BPA complied, n (%)	Order complied, n (%)	Administration complied, n (%)
**Age (years)**						
	<40	31,301	1576 (5)	956 (60.7)	1019 (64.7)	1169 (74.2)
	40-60	49,464	3567 (7.2)	2180 (61.1)	2421 (67.9)	2770 (77.7)
	60-80	74,532	6528 (8.8)	3842 (58.9)	4350 (66.6)	5065 (77.6)
	>80	22,800	2636 (11.6)	1571 (59.6)	1827 (69.3)	2100 (79.7)
**Sex**						
	Female	93,681	7208 (7.7)	4293 (59.6)	4845 (67.2)	5533 (76.8)
	Male	84,416	7099 (8.4)	4256 (60.0)	4772 (67.2)	5571 (78.5)
**Race**						
	Caucasian	132,568	10,848 (8.2)	6387 (58.9)	7303 (67.3)	8420 (77.6)
	Black	17,264	1501 (8.7)	966 (64.4)	1000 (66.6)	1150 (76.6)
	Other race	28,265	1958 (6.9)	1196 (61.1)	1314 (67.1)	1534 (78.3)
**Ethnicity**						
	Hispanic	26,761	1820 (6.8)	1094 (60.1)	1207 (66.3)	1394 (76.6)
	Non-Hispanic	151,336	12,487 (8.3)	7455 (59.7)	8410 (67.4)	9710 (77.8)
**Location**						
	ICU^b^	35,011	4021 (11.5)	2244 (55.8)	2609 (64.9)	3079 (76.6)
	Telemetry	70,804	7793 (11)	4601 (59)	5200 (66.7)	6027 (77.3)
	OB/Gyn^c^	1044	14 (1.3)	8 (57.1)	7 (50)	9 (64.3)
	Stepdown	29,082	3534 (12.2)	2048 (58)	2353 (66.6)	2709 (76.7)
**Medications**						
	Azithromycin	15,428	1565 (10.1)	1093 (69.8)	1214 (77.6)	1312 (83.8)
	Citalopram	3669	465 (12.7)	76 (16.3)	237 (51)	286 (61.5)
	Droperidol	6556	346 (5.3)	205 (59.2)	193 (55.8)	217 (62.7)
	Escitalopram	4956	635 (12.8)	87 (13.7)	306 (48.2)	353 (55.6)
	Haloperidol	26,842	2271 (8.5)	1397 (61.5)	1622 (71.4)	1994 (87.8)
	Hydroxychloroquine	1299	94 (7.2)	17 (18.1)	42 (44.7)	49 (52.1)
	Levofloxacin	9581	928 (9.7)	562 (60.6)	640 (69)	775 (83.5)
	Methadone	1577	196 (12.4)	51 (26)	93 (47.4)	101 (51.5)
	Ondansetron	107,236	7202 (6.7)	4997 (69.4)	5152 (71.5)	5863 (81.4)
	Sotalol	953	605 (63.5)	64 (10.6)	118 (19.5)	154 (25.5)
**Cluster**						
	0	8609	502 (5.8)	351 (69.9)	392 (78.1)	410 (81.7)
	1	46,258	4044 (8.7)	2230 (55.1)	2649 (65.5)	3126 (77.3)
	2	12,981	1731 (13.3)	982 (56.7)	1122 (64.8)	1338 (77.3)
	3	110,249	8030 (7.3)	4986 (62.1)	5454 (67.9)	6230 (77.6)
	Total	178,097	14,963 (84)	8549 (57.1)	9617 (64.3)	11,104 (74.2)

^a^BPA: BestPractice Advisory.

^b^ICU: intensive care unit.

^c^OB/Gyn: obstetrics and gynecology.

### Impact of Alert on Outcomes

Across all medications, the probability of diLQTS was 16.7% (SD 0.6) if the BPA alert fired versus 8.5% (SD 0.1) if the alert did not fire (OR 2.28, 95% CI 2.10-2.47; *P*<.001 for adjusted model without interactions; Table S3A in Multimedia Appendix 1). Location made a difference in risk, as ICU patients in whom the BPA fired were at lower risk for diLQTS than those without the alert (OR 0.76, 95% CI 0.65-0.89; *P*<.001 for adjusted model with ICU-alert interaction; Table S3B in Multimedia Appendix 1), as were patients on telemetry (OR 0.72, 95% CI 0.61-0.85; *P*<.001 for adjusted model with telemetry-alert interaction; Table S3C in Multimedia Appendix 1). Although individual medications were individually associated with the risk of diLQTS (Table S3A in Multimedia Appendix 1), there was no evidence of a significant impact of the alert on diLQTS for any specific medication (Table S3D in Multimedia Appendix 1) or across all (*P*=.88). Hospitalization duration was significantly longer in patients in whom the alert fired (incidence rate ratio 1.08, 95% CI 1.07-1.09; *P*<.001; Table S4 in Multimedia Appendix 1). Inpatient mortality was significantly higher in those who had the alert fire (OR 1.33, 95% CI 1.19-1.49; *P*<.001; Table S5A in Multimedia Appendix 1), although we did not note a difference in ICU (OR 0.92, 95% CI 0.70-1.21; *P*=.56; Table S5B in Multimedia Appendix 1), telemetry (OR 0.83, 95% CI 0.59-1.18; *P*=.30; Table S5C in Multimedia Appendix 1), or across medications (*P*=.30; Table S5D in Multimedia Appendix 1). The alert was also associated with increased 3-month (OR 1.30, 95% CI 1.20-1.42; *P*<.001; Table S6 in Multimedia Appendix 1), 6-month (OR 1.22, 95% CI 1.14-1.31; *P*<.001; Table S7 in Multimedia Appendix 1), and 1-year mortality (OR 1.14, 95% CI 1.07-1.20; *P*<.001; Table S8 in Multimedia Appendix 1).

Within the BPA, there was no evidence of an effect of provider actions on the risk of diLQTS overall (*P*=.79; Table S9A in Multimedia Appendix 1), or for any specific medication (Table S9A in Multimedia Appendix 1), as well as no difference in inpatient (*P*=.18; Table S9B in Multimedia Appendix 1), 3-month (*P*=.25; Table S9C in Multimedia Appendix 1), 6-month (*P*=.34; Table S9D in Multimedia Appendix 1), or 1-year (*P*=.21; Table S9E in Multimedia Appendix 1) mortality. However, we did note a medication-specific impact on hospital duration with provider compliance (*P*<.001; Table S9F in Multimedia Appendix 1), with the biggest impact in those treated with levofloxacin (IRR 1.50, 95% CI 1.39-1.62; *P*<.001; Table S9F in Multimedia Appendix 1), indicating that hospital duration was 50% longer in those patients where the provider complied with recommendations from the alert. These results suggest that alert response had either minimal clinical impact, or resulted in longer hospital stay, across medications after adjustment for other comorbidities.

### CDS Implementation

Table 3 lists the number of medication orders before and after the CDS was deployed for each medication. After adjustment for age, sex, race and ethnicity, location, laboratory findings, medication, and comorbidity clusters, there was no difference in risk of diLQTS based on CDS deployment (OR 1.13, 95% CI 0.94-1.36; *P*=.21; Table S10A in Multimedia Appendix 1), and no evidence of effect for any individual medication (*P*=.98; Table S10B in Multimedia Appendix 1). However, we did identify an increased risk of inpatient mortality after CDS deployment (OR 1.43, 95% CI 1.09-1.88; *P*=.01; Table S11 in Multimedia Appendix 1), as well as increased 3-month (OR 1.86, 95% CI 1.49-2.33; *P*<.001; Table S12 in Multimedia Appendix 1), 6-month (OR 1.79, 95% CI 1.46-2.19; *P*<.001; Table S13 in Multimedia Appendix 1), and 1-year mortality (OR 1.79, 95% CI 1.51-2.11; *P*<.001; Table S14 in Multimedia Appendix 1). We also found that the hospital duration was shorter among those without mortality during the admission (ie, inpatient mortality) after CDS implementation than before (9.4, SD 0.01 days post CDS versus 9.7, SD 0.8 days pre-CDS, *P*<.001; Table S15 in Multimedia Appendix 1).

**Table 3:** Number of encounters before and after CDS roll-out.

Medication	Pre-CDS, n (%)	Post-CDS, n (%)
Azithromycin	477 (3.09)	14,951 (96.91)
Citalopram	239 (6.51)	3430 (93.49)
Droperidol	79 (1.21)	6477 (98.79)
Escitalopram	103 (2.08)	4853 (97.92)
Haloperidol	27 (0.1)	26,815 (99.9)
Hydroxychloroquine	86 (6.62)	1213 (93.38)
Levofloxacin	639 (6.67)	8942 (93.33)
Methadone	59 (3.74)	1518 (96.26)
Ondansetron	3233 (3.01)	104,003 (96.99)
Sotalol	13 (1.36)	940 (98.64)
Total	4955 (2.78)	173,142 (97.22)

### Impact of diLQTS on Outcomes

Among the entire population, 14,690 (8.25%) encounters had an episode of diLQTS. In a marginal Poisson model adjusted for age, sex, race and ethnicity, hospital location, cluster, and medication (interaction with diLQTS), the average hospital duration was higher among those with diLQTS compared with those who did not have diLQTS (6.18 (SD 0.02) days versus 5.40 (SD 0.01) days, *P*<.001; Table S16A in Multimedia Appendix 1). Within specific medications (Table S16A in Multimedia Appendix 1), the presence of diLQTS was associated with a statistically significant increase in hospital duration for azithromycin (5.95, SD 0.05 days vs 5.15, SD 0.12 days, *P*<.001), droperidol (5.08, SD 0.11 days vs 3.77, SD 0.03 days, *P*<.001), escitalopram (6.15, SD 0.09 vs 5.61, SD 0.03 days; *P*=.004), haloperidol (6.60, SD 0.04 vs 5.85, SD 0.01; *P*=.04), and sotalol (5.96, SD 0.02 vs 5.22, SD 0.01; *P*<.001), and no different for citalopram (*P*=.23), hydroxychloroquine (*P*=.37), levofloxacin (0.471), methadone (*P*=.15), and ondansetron (*P*=.32); Table S16B in Multimedia Appendix 1. diLQTS was not significantly associated with inpatient mortality (*P*=.866; Table S17 in Multimedia Appendix 1), 3-month mortality (*P*=.56; Table S18 in Multimedia Appendix 1), 6-month mortality (*P*=.62; Table S19 in Multimedia Appendix 1), or 1-year mortality (*P*=.27; Table S20 in Multimedia Appendix 1). There was no evidence of an inpatient mortality difference with diLQTS for any individual medication (Table S17 in Multimedia Appendix 1), although certain medications (hydroxychloroquine and ondansetron) were noted to have a paradoxical decrease in 3-, 6-, or 1-year mortality with the presence of diLQTS (see Tables S18-S20 in Multimedia Appendix 1 for details).

## Discussion

### Principal Findings

In this large-scale, system-wide observational study, we found that while the CDS system successfully identified patients at higher risk of diLQTS, the system itself had no significant impact on preventing diLQTS, regardless of how providers responded to the alerts. We found that while the risk of diLQTS itself predicted increased hospital duration, it did not have any notable effect on mortality, including inpatient and follow-up outpatient. We also found that, while hospital duration was decreased with the deployment of the CDS tools, inpatient and moderate-term outpatient mortality were actually increased after the deployment of the CDS. Taken in their entirety, these results suggest that despite accurately predicting patients at risk for diLQTS, the CDS system deployed from this risk prediction did not reduce the risk of diLQTS, its intended goal.

As noted in the Introduction, it is generally assumed that the critical component of many CDS tools designed for risk mitigation is their ability to accurately predict risk in individual patients. Our group, and others, have proposed that the integration of more sophisticated models, such as using deep learning or other machine learning algorithms [9,10], or the inclusion of additional predictors, such as genetic risk scores [7], might improve the predictive accuracy of an alert system for diLQTS. Yet, while these approaches could in theory improve the accuracy of identifying patients at high risk of diLQTS, our findings do not suggest that identification of high-risk individuals was a key limitation of the current system. Patients in whom the BPA fired had a significantly higher (>3-fold) risk of diLQTS, including after adjustment for specific medications and other clinical risk factors. While the specific risk estimate could theoretically be improved with these additional integrations, it does not seem that the present system failed to identify patients at high risk.

And yet, despite the alert firing in appropriately high-risk individuals, the impact on clinical outcomes, including diLQTS, was not significantly impacted. There was no difference in diLQTS before or after the CDS tools were implemented, and provider response within or subsequent to the BPA did not impact the risk of diLQTS. Mortality was actually increased after the CDS tools were implemented, although we suspect this finding to represent confounding by unmeasured patient or treatment factors rather than a mechanism causally related to the CDS itself (see below). Regardless, provider adherence to the BPA, whether through actions within the alert, medication orders, or administration of the culprit medication, did not impact any hard clinical outcomes. In plain terms, we found no evidence that the CDS used within the system was improving patient safety or outcomes.

While numerous published studies have suggested the efficacy of CDS to prevent diLQTS, closer inspection reveals that few systematically evaluated the outcome of diLQTS itself in their evaluation. Gallo et al [1] examined a CDS using the modified Tisdale QT risk score and noted an impact on provider actions, although the impact on diLQTS was not evaluated. Tisdale et al [4] also examine the risk score for diLQTS in a CDS, and noted a change in prescriber patterns, but did not assess diLQTS [5]. Other groups [12] have performed similar evaluations of CDS for diLQTS focused on provider actions without an examination of diLQTS itself, including the CDS presently deployed within our system [13,14]. Such a surrogate measure of CDS effectiveness is common, and while it is not unreasonable to consider provider actions as a surrogate for clinical outcomes, these cases are a reminder that there are limitations to drawing inference from surrogate outcomes alone [15], a finding that was suggested in our prior work [11] and is confirmed in this investigation.

In terms of the evaluation of CDS systems broadly, it cannot be overstated how our findings highlight the need to use additional methods and frameworks beyond a simple inspection of discrimination and calibration. Specifically, there is a well-established field of implementation science that includes methods such as the RE-AIM (Reach, Effectiveness, Adoption, Implementation, Maintenance) framework [16,17], which includes activities seeking an evaluation of stakeholder engagement and methods to address barriers to implementation. In addition to the inclusion of a formal process to categorize and process user feedback, these methods also frequently use experts from behavioral psychology to understand the context in which a provider might choose to ignore an alert or situations in which the alert may not even have been acknowledged in the first place. Such efforts might have highlighted the relatively low rate of compliance with the QT alert and potentially led to improvements in design and activation prior to noting an overall lack of clinical impact. These insights will serve as a guide in future work by our team to use implementation science methods to refine and improve the QT CDS.

There are several key limitations to this investigation, the predominant being that the impact of the CDS was studied using observational data rather than through a prospective clinical trial. Like any observational study, there was a high risk of confounding, even with the inclusion of clinical risk factors such as other medications, diagnoses, or patient location (a marker of disease severity). We are skeptical that the CDS itself was likely to have directly increased patient mortality as we identified and suspect that this finding is one example of this limitation. However, this limitation highlights a growing problem with CDS tool development and implementation, which is that many tools are inconsistently evaluated and rarely evaluated prospectively prior to widespread implementation. Particularly for a condition like diLQTS, the argument is made that since prospective studies would likely be underpowered to detect a meaningful difference in clinical outcomes, and since the downsides of simply “alerting” providers that a patient is high risk would seem negligible in contrast with a new medication or procedure, then it is sufficient to forego formal prospective testing as long as the CDS “seems reasonable.” However, with increasing attention to the challenges of alert fatigue [18,19], these assumptions do not seem sufficient to justify empirical implementation of these alert systems. Especially in situations where the frequency of alerts is anticipated to be high, a plan for evaluation at a defined period is key to tackling issues that could result in alert fatigue.

### Limitations

Some additional considerations of these results should also be identified. First, the alerts studied were all applied for inpatients, which might indicate that providers in general were more aware of risks of diLQTS and other adverse outcomes to a greater degree than in prescribing medications for outpatients. Interestingly, patients in the ICU and on telemetry were less likely to have diLQTS if the alert fired, suggesting that if they were in a setting where medications could be titrated or monitored more closely, then the risks could be mitigated. In contrast, one could surmise that a CDS tool deployed in the outpatient setting could have a greater impact, as providers may be more likely to make changes to medications to avoid diLQTS in a setting where monitoring is less available. The CDS in our system has also been deployed in the outpatient setting, and we plan to perform a similar investigation in that population as well in future studies.

Second, a potential confounder of outcome adjudication in this study could have been increased surveillance of ECG monitoring after the alert fired, a finding noted by Gallo et al [1], as well. In this case, those patients in whom the BPA fired may have been more likely to have follow-up ECGs where diLQTS could have been diagnosed. We did not have a simple metric to assess enhanced surveillance following the alert, which is relevant since, particularly for medications where there was no alternative available, this would be the recommended action. Some medications, such as sotalol [20], actually require ECGs to be performed after each dose to monitor for diLQTS, and our finding that this medication carried a high risk of diLQTS could potentially be explained by the increased monitoring (more ECGs) among these patients.

Third, the actual risk of torsade de pointes, even among patients with diLQTS, is still quite low, and although the adopted definition of diLQTS is based on corrected QT intervals greater than 500 milliseconds, it is possible that a mortality risk of diLQTS (in the form of TdP) is not present unless the QT interval is much longer or if there are additional factors such as sinus pauses or severe bradycardia immediately preceding the TdP event. Further, there are several well-established methods to calculate the corrected QT interval, such as those of Bazett [21] or Sagie [22], which could have a differential impact on the prediction of TdP. Vandenberk et al [23] noted that Fridericia, as used in this study, is preferred over Bazett’s for appropriate correction to predict mortality, and although we do not suspect that our results would be heavily influenced based on the method of correction, we acknowledge that the population-level definition of diLQTS may not reflect the individual risk of TdP; other methods to find the appropriate degree of QT prolongation leading to TdP may be needed to appropriately evaluate the impact of these tools.

### Conclusions

In conclusion, we found that although a simple, rule-based algorithm can identify patients at high risk of diLQTS, a CDS tool created from this algorithm did not demonstrate a system-wide change in risk of diLQTS or other clinical outcomes. We believe these findings provide support for the need for evaluation and ideally randomized, comparative trials of CDS tools when possible.

## Appendix

Multimedia Appendix 1 Additional material.

## Abbreviations

BPA: BestPractice Advisory
CDS: clinical decision support
diLQTS: drug-induced QT prolongation
ECG: electrocardiograph
EHR: electronic health record
ICD-9: International Classification of Diseases, Ninth Revision
ICD-10: International Statistical Classification of Diseases, Tenth Revision
ICU: intensive care unit
OB/Gyn: obstetrics/gynecology floor
OR: odds ratio
QTc: heart rate–corrected QT interval
RE-AIM: Reach, Effectiveness, Adoption, Implementation, Maintenance
