# Supplementary material for: Impact of an Alert-Based Inpatient Clinical Decision Support Tool to Prevent Drug-Induced Long QT Syndrome: Large-Scale, System-Wide Observational Study
Source: J Med Internet Res. 2025 Apr 14;27:e68256. doi: 10.2196/68256 (PMC12038287; doi:10.2196/68256)
Supplement: Multimedia Appendix 1 [file jmir_v27i1e68256_app1.pdf]

Supplemental Material

Supplemental Methods: Page 2
Supplemental Figures: Page 3
Supplemental Tables: Pages 4-36

Supplemental Methods

Data extraction process: Data was collected entirely retrospectively, with extraction conducted using the Health Date Compass EHR repository, an institutional resource containing a full copy of the EHR backend for research and analysis. The repository is a structured database, queried using SQL to obtain the dataset for this analysis. Our goal in the extraction process for this investigation was twofold. First, to obtain a dataset with all patients in whom the BPA fired for each culprit medication, from which we could evaluate the impact of providers' responses on risk of diLQTS, hospital duration, inpatient mortality, and subsequent 3-, 6-, and 1-year mortality. Second, we sought to create a dataset for all inpatients in whom the culprit medications were ordered, regardless of QT interval or whether a BPA fired, to examine the impact of the CDS on these outcomes. Acknowledging the importance of temporal relationships throughout the hospital course, we focused our extraction on specific determination of events during this time period. We outline this process in detail below.

For all patients, the extraction process started with selection of the timestamp, encounter ID, and unique person ID for each encounter (hospitalization) where an electronic order was placed (entered or signed) for a culprit medication at any time during the hospitalization. The first order for each culprit medication was used, such that an individual patient could have multiple entries if multiple culprit medications were prescribed on a given encounter, but only one entry per culprit medication per encounter. If the BPA alert fired at the time of the order entry for the culprit medication, an indicator variable was created for this event (yes/no) as well as the timestamp. Among these patients, if the provider then signed the order for the medication (meaning they chose to override the alert), this order was also timestamped along with an indicator of this specific action among those in whom the BPA fired. In this manner, the timestamp of the medication order could correspond to the initial order if the BPA alert did not fire, or the follow-up action by the provider if the BPA alert did fire. We then extracted the administration timing from the nursing record for whether the patient was actually administered the medication after the order was placed. The administration record was thus recorded for all medications ordered, regardless of whether the alert fired or not.

To establish the temporal relationships among events in the medical record, we first established a timestamp for the hospital admission (date/time patient was formally admitted to the hospital) as time_0. The order_time was then calculated as the time in hours between the time_0 and the electronic order 'signed' of the culprit medication. The CDS timestamp was assigned as the time interval (in hours) between time_0 and the CDS alert for those subjects in whom it fired. The admin_time is the time interval between time_0 and documented administration of the culprit medication in the nursing chart. The lab_time was calculated for potassium, magnesium, and creatinine labs as the time between the reported values of these labs in the chart and time_0. Hospital_duration was defined as the interval in hours between time_0 and either discharge or death.

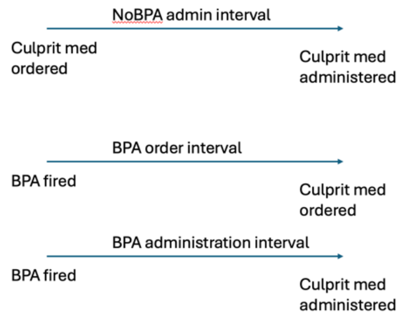

Figure 1. Derived time intervals. Based on relative timing of medication order and BPA firing.

From these intervals, we derived the following additional intervals (Fig. 1). For subjects in whom the BPA did not fire, the time interval in hours was recorded between the time of the medication order and the time of administration. Meds administered prior to the order, or those administered more than 48 hours after the order were recoded to missing. For subjects in whom the BPA fired, the time interval between the subsequent medication order was derived in hours, and filtered to recode as missing those with the order prior to the alert. In these subjects, an additional interval was calculated as the time between the BPA alert and medication administration, with recoding for those administered the medication prior to the alert. Lab times were coded based on timing relative to the medication administration, and documented first administration was used as the time index for determination of effects of the medication on the QTc.

Supplemental Figures

Supplemental Figure 1A (top). Inertia scores across cluster numbers (k). Elbow method identifies k=4 as reasonable.
Supplemental Figure 1B (bottom). PCA analysis with cluster labels.

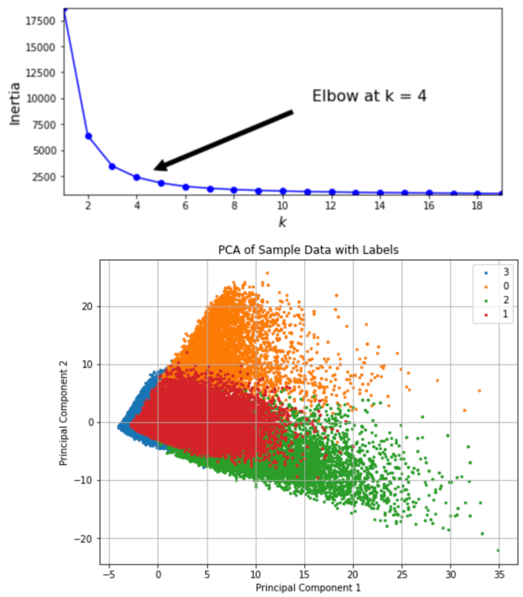

Supplemental Tables

Supplemental Table 1. Most common medications and diagnoses by cluster.				
	**0**	**1**	**2**	**3**
**Meds**	Naloxone IV (N=8460)	Ondansetron IV (N=34099)	Magnesium sulfate IV (N=10847)	Ondansetron IV (N=21038)
	Fentanyl IV (N=7952)	Acetaminophen PO (N=29399)	Potassium chloride IV (N=9695)	Normal saline IV (N=14055)
	Ondansetron IV (N=7918)	Melatonin PO (N=21633)	Acetaminophen PO (N=8552)	Lactated ringer's IV (N=11683)
	Acetaminophen PO (N=7582)	Polyethylene glycol PO (N=17407)	Ondansetron IV (N=7450)	Ondansetron PO (N=11674)
	Haloperidol IV (N=7517)	Ondansetron PO (N=16528)	Polyethylene glycol IV (N=5994)	Fentanyl IV (N=11510)
	Glucagon IV (N=6961)	Oxycodone (N=14951)	Potassium phosphate IV (N=5536)	Acetaminophen PO (N=10643)
	Insulin Lispro IV (N=6935)	Sennosides PO (N=13984)	Bisacodyl PR (N=5175)	Lidocaine Injection (N=7245)
	Oxycodone PO (N=6886)	Bisacodyl PR (N=13684)	Sennosides PO (N=5028)	Midazolam IV (N=6124)
	Dextrose IV (N=6866)	Dextrose IV (N=11763)	Dextrose IV (N=4819)	Oxycodone PO (N=5764)
	Prochlorperazine IV (N=6744)	Insulin Lispro IV (N=11241)	Magnesium oxide PO (N=4593)	Melatonin PO (N=5114)
**Diagnoses**				
	Anemia (N=549)	Hypertension (N=3091)	Acute respiratory failure (N=1612)	Hypoxemia (N=3276)
	Obesity (N=528)	Anemia (N=3032)	Anemia (N=1583)	Pneumonia (N=3269)
	Esophageal reflux (N=466)	Pneumonia (N=2995)	Hypertension (N=1067)	Hypertension (N=3037)
	Hypertension (N=422)	Hypoxemia (N=2973)	Sepsis (N=903)	Acute kidney failure (2563)
	History of TIA/stroke (N=351)	Sepsis (N=2820)	Acute kidney failure (N=899)	Sepsis (N=2364)
	Renal/ureteral disease NOS (N=305)	Acute kidney failure (N=2632)	Thrombocytopenia (N=735)	Acute respiratory failure (N=2311)
	Diabetes mellitus type II (N=275)	Acute respiratory failure (N=2525)	Hypokalemia (N=647)	Anemia (N=2124)
	Fracture of femoral neck (N=262)	Hyponatremia (N=1984)	Hyponatremia (N=623)	Hyponatremia (N=2095)
	Atrial fibrillation (N=258)	Esophageal reflux (N=1713)	Obesity (N=599)	Chest pain (N=2048)
	Acute kidney failure (N=258)	Other electrolyte abnormality (N=1701)	Pneumonia (N=588)	Nausea (N=2032)

**Supplemental Table 2. Diagnoses that were more common and less common in each cluster compared with other clusters.**		
**Cluster**	**Most common**	**Least common**
**0**	History of TIA/stroke	Acute respiratory failure
	Obesity	Pneumonia
	Post-procedure state	Sepsis
	GERD	Viral infection
	Kidney/ureteral disease NOS	Acidosis
	Femoral neck fracture	Encephalopathy
	Hypothyroidism	Chest pain
	Aortic stenosis	
**1**	Pneumonia	None
	Hypoxemia	
	Sepsis	
	Electrolyte abnormality NOS	
	Chest pain	
	Fatigue/malaise	
	Weakness	
	COPD	
**2**	Acute respiratory failure	Femur fracture
	Anemia	Aortic stenosis
	Hypertension	Car injury
	Sepsis	Kidney transplant
	Thrombocytopenia	Abdominal pain
	Acute kidney failure	Intestinal obstruction
	Hypokalemia	Acute cholecystitis
	Hyponatremia	
**3**	Vomiting	Anemia
	Dizziness	Hypertension
		Sepsis
		Thrombocytopenia
		Acute kidney failure
		Hypokalemia
		Hyponatremia

Supplemental Table 3A. Multivariable marginal logistic regression model for drug-induced QT prolongation (diLQTS) without interaction terms. bpa_fired = alert fired after medication order; admin_yn = medication was administered irrespective of alert firing.

```
GEE population-averaged model                   Number of obs    =  63,858
Group variable: arb_personid                    Number of groups =  39,381
Family: Binomial                                Obs per group:
Link:   Logit                                                min =       1
Correlation: exchangeable                                    avg =     1.6
                                                             max =      31
                                                Wald chi2(30)    = 1579.39
Scale parameter = 1                             Prob > chi2      =  0.0000
```

dilqts	Odds ratio	Std. err.	z	P>|z|	[95% conf.	interval]
age	1.000639	.0009758	0.66	0.512	.9987287	1.002554
female	1.004069	.0321342	0.13	0.899	.943022	1.069068
maxpreqtc	1.001357	.0002562	5.30	0.000	1.000855	1.001859
race_caucasian	.9640599	.0503222	-0.70	0.483	.8703076	1.067911
race_black	.9766925	.070089	-0.33	0.742	.8485439	1.124194
ethnicity_hisp	.9566644	.052635	-0.81	0.421	.8588693	1.065595
icu	1.768316	.0711116	14.17	0.000	1.634291	1.913332
tele	1.139796	.0456271	3.27	0.001	1.053787	1.232825
ob	.4968545	.1623188	-2.14	0.032	.2619074	.9425637
stepdown	1.230058	.0402584	6.33	0.000	1.153631	1.311549
cluster_4						
1	1.055774	.0826712	0.69	0.488	.9055626	1.230902
2	1.178773	.0969867	2.00	0.046	1.003218	1.385049
3	1.203847	.0939836	2.38	0.017	1.033043	1.402892
magnesium_time	.9999673	.0001168	-0.28	0.779	.9997384	1.000196
magnesium_value	1.339222	.0461388	8.48	0.000	1.251777	1.432775
potassium_interval	.9996162	.0003725	-1.03	0.303	.9988863	1.000347
potassium_value	.8011637	.0181134	-9.81	0.000	.7664372	.8374637
creat_interval	1.000411	.0003795	1.08	0.279	.9996673	1.001155
creatinine_value	1.005615	.0086556	0.65	0.515	.9887929	1.022724
1.admin_yn	1.043872	.0304546	1.47	0.141	.9858563	1.105301
1.bpa_fired	2.277252	.0931967	20.11	0.000	2.101723	2.46744
med						
CITALOPRAM	1.222742	.1070902	2.30	0.022	1.029876	1.451726
DROPERIDOL	1.165461	.0995913	1.79	0.073	.9857358	1.377954
ESCITALOPRAM	1.084829	.0842202	1.05	0.294	.9317056	1.263118
HALOPERIDOL	1.072724	.0595299	1.27	0.206	.9621687	1.195982
HYDROXYCHLOROQUINE	1.341883	.1675727	2.35	0.019	1.050552	1.714003
LEVOFLOXACIN	.8762935	.0580464	-1.99	0.046	.7696004	.9977778
METHADONE	1.377255	.1464249	3.01	0.003	1.118196	1.696334
ONDANSETRON	1.116686	.0511906	2.41	0.016	1.02073	1.221663
SOTALOL	2.23606	.241033	7.47	0.000	1.810212	2.762088
_cons	.0380172	.006905	-18.00	0.000	.0266304	.0542727

Note: **_cons** estimates baseline odds (conditional on zero random effects).

Supplemental Table 3B. Multivariable marginal logistic regression model for drug-induced QT prolongation (diLQTS) with interaction term for intensive care unit (ICU) with alert. bpa_fired = alert fired after medication order; admin_yn = medication was administered irrespective of alert firing.

```
GEE population-averaged model                   Number of obs     =  63,858
Group variable: arb_personid                    Number of groups =  39,381
Family: Binomial                                Obs per group:
Link:   Logit                                                min =       1
Correlation: exchangeable                                    avg =     1.6
                                                             max =      31
                                                Wald chi2(31)    = 1573.81
Scale parameter = 1                             Prob > chi2      =  0.0000
```

dilqts	Odds ratio	Std. err.	z	P>|z|	[95% conf. interval]	
age	1.000621	.0009754	0.64	0.524	.9987113	1.002535
female	1.004748	.0321438	0.15	0.882	.9436814	1.069765
maxpreqtc	1.001355	.000256	5.30	0.000	1.000854	1.001857
race_caucasian	.9654481	.0503777	-0.67	0.500	.8715909	1.069412
race_black	.9772352	.0701134	-0.32	0.748	.8490401	1.124786
ethnicity_hisp	.9578471	.0526802	-0.78	0.434	.8599661	1.066869
tele	1.132952	.0455091	3.11	0.002	1.047176	1.225753
ob	.5009346	.1637235	-2.12	0.034	.2639841	.9505706
stepdown	1.2296	.0402016	6.32	0.000	1.153278	1.310973
cluster_4						
1	1.05813	.0828754	0.72	0.471	.9075505	1.233694
2	1.182474	.0972756	2.04	0.042	1.006394	1.389361
3	1.202018	.0938777	2.36	0.018	1.031412	1.400845
magnesium_time	.9999762	.0001164	-0.20	0.838	.9997481	1.000204
magnesium_value	1.338118	.0460768	8.46	0.000	1.250789	1.431544
potassium_interval	.9996182	.0003719	-1.03	0.305	.9988895	1.000347
potassium_value	.8026662	.018148	-9.72	0.000	.7678734	.8390354
creat_interval	1.000401	.0003789	1.06	0.289	.999659	1.001144
creatinine_value	1.005369	.0086701	0.62	0.535	.9885188	1.022507
1.icu	1.851203	.0779091	14.63	0.000	1.704633	2.010377
1.bpa_fired	2.634236	.1489649	17.13	0.000	2.357868	2.942996
icu#bpa_fired						
1 1	.7572364	.0580697	-3.63	0.000	.6515623	.8800493
1.admin_yn	1.047134	.0305494	1.58	0.114	.9889381	1.108755
med						
CITALOPRAM	1.217333	.1066615	2.24	0.025	1.025246	1.445409
DROPERIDOL	1.167416	.0998805	1.81	0.070	.9871871	1.380549
ESCITALOPRAM	1.083102	.0840957	1.03	0.304	.9302064	1.261129
HALOPERIDOL	1.077235	.0597943	1.34	0.180	.96619	1.201042
HYDROXYCHLOROQUINE	1.339756	.1673544	2.34	0.019	1.048814	1.711405
LEVOFLOXACIN	.8786965	.0582194	-1.95	0.051	.7716872	1.000545
METHADONE	1.367879	.1454552	2.95	0.003	1.11054	1.68485
ONDANSETRON	1.119321	.051342	2.46	0.014	1.023083	1.224611
SOTALOL	2.123157	.2306496	6.93	0.000	1.715978	2.626955
_cons	.036975	.0067207	-18.14	0.000	.0258935	.052799

Note: **_cons** estimates baseline odds (conditional on zero random effects).

Supplemental Table 3C. Multivariable marginal logistic regression model for drug-induced QT prolongation (diLQTS) with interaction term for telemetry monitoring with alert. bpa_fired = alert fired after medication order; admin_yn = medication was administered irrespective of alert firing.

```
GEE population-averaged model                  Number of obs    =  63,858
Group variable: arb_personid                   Number of groups =  39,381
Family: Binomial                               Obs per group:
Link:   Logit                                                min =       1
Correlation: exchangeable                                    avg =     1.6
                                                             max =      31
                                               Wald chi2(31)    = 1578.03
Scale parameter = 1                            Prob > chi2      =  0.0000
```

dilqts	Odds ratio	Std. err.	z	P>|z|	[95% conf.	interval]
age	1.000628	.0009756	0.64	0.520	.9987178	1.002542
female	1.004332	.0321338	0.14	0.893	.9432853	1.06933
maxpreqtc	1.001354	.000256	5.29	0.000	1.000852	1.001856
race_caucasian	.9649111	.0503509	-0.68	0.494	.871104	1.06882
race_black	.9745811	.0699406	-0.36	0.720	.8467041	1.121771
ethnicity_hisp	.9576438	.0526706	-0.79	0.431	.8597808	1.066646
icu	1.765001	.0708503	14.15	0.000	1.631459	1.909474
1.tele	1.19605	.0501025	4.27	0.000	1.101774	1.298393
1.bpa_fired	2.888113	.210551	14.55	0.000	2.503568	3.331724
tele#bpa_fired						
1 1	.7241124	.0605531	-3.86	0.000	.6146462	.8530742
ob	.5033881	.1644768	-2.10	0.036	.2653271	.9550458
stepdown	1.229348	.0402023	6.31	0.000	1.153025	1.310723
cluster_4						
1	1.059794	.0830363	0.74	0.459	.9089266	1.235704
2	1.185644	.097588	2.07	0.039	1.009005	1.393205
3	1.207188	.0943112	2.41	0.016	1.035798	1.406938
magnesium_time	.9999739	.0001165	-0.22	0.823	.9997455	1.000202
magnesium_value	1.337921	.0460964	8.45	0.000	1.250557	1.431389
potassium_interval	.9996164	.0003724	-1.03	0.303	.9988867	1.000347
potassium_value	.802456	.0181451	-9.73	0.000	.7676688	.8388197
creat_interval	1.000408	.0003794	1.08	0.282	.9996649	1.001152
creatinine_value	1.005176	.0086932	0.60	0.551	.9882816	1.02236
1.admin_yn	1.046733	.0305398	1.57	0.117	.9885552	1.108334
med						
CITALOPRAM	1.217986	.1067161	2.25	0.024	1.0258	1.446178
DROPERIDOL	1.1659	.0997388	1.79	0.073	.9859255	1.378729
ESCITALOPRAM	1.08731	.0844126	1.08	0.281	.9338366	1.266006
HALOPERIDOL	1.076536	.0597509	1.33	0.184	.9655717	1.200253
HYDROXYCHLOROQUINE	1.344424	.1679065	2.37	0.018	1.052516	1.71729
LEVOFLOXACIN	.8791962	.0582536	-1.94	0.052	.772124	1.001116
METHADONE	1.372669	.1459628	2.98	0.003	1.114432	1.690746
ONDANSETRON	1.119598	.0513413	2.46	0.014	1.023361	1.224886
SOTALOL	2.168796	.2342548	7.17	0.000	1.755008	2.680144
_cons	.0364499	.0066328	-18.20	0.000	.0255154	.0520704

Note: **_cons** estimates baseline odds (conditional on zero random effects).

Supplemental Table 3D. Multivariable marginal logistic regression model for drug-induced QT prolongation (diLQTS) with interaction term for medication with alert. Note that the P value for marginal linear predictors across all meds (degrees of freedom = 9) was 0.877 (Chi-square = 4.49). bpa_fired = alert fired after medication order; admin_yn = medication was administered irrespective of alert firing.

```
GEE population-averaged model                   Number of obs    =  63,858
Group variable: arb_personid                    Number of groups =  39,381
Family: Binomial                                Obs per group:
Link:   Logit                                                min =       1
Correlation: exchangeable                                    avg =     1.6
                                                             max =      31
                                                Wald chi2(39)    = 1581.64
Scale parameter = 1                             Prob > chi2      =  0.0000
```

dilqts	Odds ratio	Std. err.	z	P>|z|	[95% conf.	interval]
age	1.000652	.0009761	0.67	0.504	.9987404	1.002567
female	1.00422	.0321497	0.13	0.895	.9431435	1.069251
maxpreqtc	1.001357	.0002562	5.30	0.000	1.000854	1.001859
race_caucasian	.9633351	.0502892	-0.72	0.474	.8696448	1.067119
race_black	.9764436	.0700755	-0.33	0.740	.8483202	1.123918
ethnicity_hisp	.9561818	.0526163	-0.81	0.415	.8584222	1.065075
icu	1.77008	.0712116	14.19	0.000	1.635868	1.915302
tele	1.138768	.0456016	3.25	0.001	1.052808	1.231747
ob	.4979393	.1626916	-2.13	0.033	.2624602	.94469
stepdown	1.230174	.0402754	6.33	0.000	1.153716	1.3117
cluster_4						
1	1.059048	.0829645	0.73	0.464	.9083094	1.234804
2	1.183318	.0974304	2.04	0.041	1.00697	1.39055
3	1.206606	.0942358	2.40	0.016	1.035349	1.406191
magnesium_time	.9999674	.0001167	-0.28	0.780	.9997386	1.000196
magnesium_value	1.339622	.0461611	8.49	0.000	1.252135	1.433221
potassium_interval	.9996217	.0003735	-1.01	0.311	.9988899	1.000354
potassium_value	.8008953	.0181146	-9.82	0.000	.7661668	.8371978
creat_interval	1.000408	.0003804	1.07	0.283	.9996631	1.001154
creatinine_value	1.005628	.0086485	0.65	0.514	.9888198	1.022723
1.admin_yn	1.04622	.0306302	1.54	0.123	.9878762	1.10801
1.bpa_fired	2.529061	.3426563	6.85	0.000	1.939242	3.298272
med						
CITALOPRAM	1.27079	.122356	2.49	0.013	1.052245	1.534724
DROPERIDOL	1.190266	.1064216	1.95	0.051	.9989373	1.418241
ESCITALOPRAM	1.079343	.0934545	0.88	0.378	.910874	1.27897
HALOPERIDOL	1.101178	.0648029	1.64	0.101	.9812179	1.235804
HYDROXYCHLOROQUINE	1.370648	.1833306	2.36	0.018	1.054566	1.781468
LEVOFLOXACIN	.8664124	.0614813	-2.02	0.043	.7539156	.9956957
METHADONE	1.337003	.161182	2.41	0.016	1.05564	1.693358
ONDANSETRON	1.129892	.0548466	2.52	0.012	1.02735	1.242669
SOTALOL	2.438536	.4812584	4.52	0.000	1.656303	3.590198
bpa_fired#med						
1#CITALOPRAM	.8009099	.1814665	-0.98	0.327	.513715	1.248662
1#DROPERIDOL	.8136917	.2335736	-0.72	0.473	.4635738	1.428239
1#ESCITALOPRAM	.9638046	.1931167	-0.18	0.854	.6507803	1.427393
1#HALOPERIDOL	.804377	.1382196	-1.27	0.205	.574373	1.126485
1#HYDROXYCHLOROQUINE	.8480487	.2988129	-0.47	0.640	.4251057	1.691783
1#LEVOFLOXACIN	1.051758	.2081431	0.25	0.799	.7136128	1.550132
1#METHADONE	1.067039	.2751921	0.25	0.801	.643655	1.768917
1#ONDANSETRON	.8978237	.1302651	-0.74	0.458	.6756011	1.193141
1#SOTALOL	.8168687	.2090522	-0.79	0.429	.4946678	1.348935
_cons	.0374488	.0068283	-18.01	0.000	.0261958	.0535358

Note: **_cons** estimates baseline odds (conditional on zero random effects).

Supplemental Table 4. Multivariable marginal Poisson model for hospital duration without interactions. bpa_fired = alert fired after medication order; admin_yn = medication was administered irrespective of alert firing; IRR = incidence-rate ratio.

```
GEE population-averaged model                   Number of obs     =     63,734
Group variable: arb_personid                    Number of groups  =     39,308
Family: Poisson                                 Obs per group:
Link:   Log                                                   min =          1
Correlation: exchangeable                                     avg =        1.6
                                                              max =         31
                                                Wald chi2(30)     =  364687.47
Scale parameter = 1                             Prob > chi2       =     0.0000
```

hospital_duration	IRR	Std. err.	z	P>|z|	[95% conf.	interval]
age	.9980802	.000093	-20.63	0.000	.997898	.9982624
female	.8931128	.002732	-36.95	0.000	.8877741	.8984836
maxpreqtc	.9998981	.0000241	-4.23	0.000	.9998509	.9999453
race_caucasian	.9327734	.0044082	-14.73	0.000	.9241735	.9414534
race_black	.9201876	.0061781	-12.39	0.000	.9081581	.9323765
ethnicity_hisp	.9997216	.0050083	-0.06	0.956	.9899536	1.009586
icu	1.591958	.0059176	125.08	0.000	1.580402	1.603599
tele	1.258376	.0050581	57.18	0.000	1.248501	1.268328
ob	1.142507	.027718	5.49	0.000	1.089452	1.198146
stepdown	1.345255	.0038434	103.81	0.000	1.337743	1.352809
cluster_4						
1	.9614747	.0061527	-6.14	0.000	.9494908	.9736098
2	1.330054	.0087686	43.26	0.000	1.312979	1.347352
3	.5972077	.0039861	-77.23	0.000	.5894459	.6050716
magnesium_time	1.000934	3.20e-06	292.18	0.000	1.000928	1.000941
magnesium_value	.9871753	.0033171	-3.84	0.000	.9806953	.9936982
potassium_interval	.9999161	.0000186	-4.52	0.000	.9998798	.9999525
potassium_value	.9758941	.0019516	-12.20	0.000	.9720765	.9797266
creat_interval	1.000697	.000019	36.65	0.000	1.000659	1.000734
creatinine_value	1.007731	.0007727	10.04	0.000	1.006217	1.009246
1.admin_yn	.8519758	.0019815	-68.88	0.000	.8481009	.8558683
1.bpa_fired	1.080836	.0045105	18.63	0.000	1.072032	1.089713
med						
CITALOPRAM	.9840226	.0079978	-1.98	0.048	.9684713	.9998236
DROPERIDOL	.890236	.0076045	-13.61	0.000	.8754555	.905266
ESCITALOPRAM	1.011428	.0066949	1.72	0.086	.9983905	1.024635
HALOPERIDOL	1.084777	.0047861	18.44	0.000	1.075436	1.094198
HYDROXYCHLOROQUINE	1.051514	.0118448	4.46	0.000	1.028553	1.074987
LEVOFLOXACIN	1.110533	.0054356	21.42	0.000	1.099931	1.121238
METHADONE	1.171649	.0100787	18.42	0.000	1.152061	1.191571
ONDANSETRON	1.052401	.0040261	13.35	0.000	1.044539	1.060321
SOTALOL	.7606628	.012499	-16.65	0.000	.7365555	.7855592
_cons	9.64096	.1576171	138.61	0.000	9.336933	9.954887

Note: **_cons** estimates baseline incidence rate (conditional on zero random effects).

Supplemental Table 5A. Multivariable marginal logistic regression model for inpatient mortality in adjusted models without interaction. bpa_fired = alert fired after medication order; admin_yn = medication was administered irrespective of alert firing

GEE population-averaged model		Number of obs =	**63,858**
Group variable: **arb_personid**		Number of groups =	**39,381**
Family: Binomial		Obs per group:	
Link: Logit		min =	**1**
Correlation: **exchangeable**		avg =	**1.6**
		max =	**31**
		Wald chi2(**30**) =	**2923.27**
Scale parameter = **1**		Prob > chi2 =	**0.0000**

inpatient_mortality	Odds ratio	Std. err.	z	P>|z|	[95% conf.	interval]
age	1.031583	.0015093	21.25	0.000	1.028629	1.034546
female	1.085446	.0455601	1.95	0.051	.999724	1.178518
maxpreqtc	1.000907	.0003365	2.70	0.007	1.000248	1.001567
race_caucasian	.9027504	.0617315	-1.50	0.135	.7895164	1.032225
race_black	.7902673	.0787752	-2.36	0.018	.650017	.9607785
ethnicity_hisp	.8485905	.0644404	-2.16	0.031	.7312393	.9847745
icu	6.324441	.4366438	26.71	0.000	5.524012	7.240853
tele	.955967	.0759001	-0.57	0.571	.8182025	1.116928
ob	.4049272	.3125664	-1.17	0.242	.0891924	1.838341
stepdown	.9903682	.0406906	-0.24	0.814	.9137427	1.073419
cluster_4						
1	3.144735	.4632397	7.78	0.000	2.356116	4.197315
2	4.296355	.640585	9.78	0.000	3.207645	5.754585
3	2.051143	.3081075	4.78	0.000	1.52804	2.753322
magnesium_time	1.000358	.0001136	3.15	0.002	1.000135	1.00058
magnesium_value	1.35741	.0614167	6.75	0.000	1.242219	1.483284
potassium_interval	.9993165	.0003569	-1.91	0.056	.9986172	1.000016
potassium_value	1.18541	.0319365	6.31	0.000	1.124439	1.249686
creat_interval	1.000174	.0003652	0.48	0.634	.9994582	1.00089
creatinine_value	1.044507	.0087764	5.18	0.000	1.027447	1.061851
1.admin_yn	.8891965	.032622	-3.20	0.001	.8275032	.9554892
1.bpa_fired	1.328266	.0771566	4.89	0.000	1.185332	1.488435
med						
CITALOPRAM	.4137098	.0562959	-6.49	0.000	.3168604	.5401615
DROPERIDOL	.6025288	.0915628	-3.33	0.001	.4473275	.8115774
ESCITALOPRAM	.4684296	.0511386	-6.95	0.000	.3781973	.5801899
HALOPERIDOL	.8424283	.0510864	-2.83	0.005	.7480222	.9487492
HYDROXYCHLOROQUINE	.8068586	.1305171	-1.33	0.185	.5876347	1.107867
LEVOFLOXACIN	.7098118	.0513002	-4.74	0.000	.6160619	.8178282
METHADONE	.6582139	.1031557	-2.67	0.008	.4841346	.8948864
ONDANSETRON	.6701686	.0342069	-7.84	0.000	.6063688	.7406813
SOTALOL	.3728871	.0930415	-3.95	0.000	.2286591	.6080877
_cons	.0002884	.0000774	-30.37	0.000	.0001705	.000488

Note: **_cons** estimates baseline odds (conditional on zero random effects).

Supplemental Table 5B. Multivariable marginal logistic regression model for inpatient mortality with ICU-alert interaction. bpa_fired = alert fired after medication order; admin_yn = medication was administered irrespective of alert firing

```
GEE population-averaged model                     Number of obs    =  63,858
Group variable: arb_personid                      Number of groups =  39,381
Family: Binomial                                  Obs per group:
Link:   Logit                                                  min =       1
Correlation: exchangeable                                      avg =     1.6
                                                               max =      31
                                                  Wald chi2(31)    = 2920.34
Scale parameter = 1                               Prob > chi2      =  0.0000
```

inpatient_mortality	Odds ratio	Std. err.	z	P>|z|	[95% conf.	interval]
age	1.031577	.0015092	21.25	0.000	1.028623	1.034539
female	1.085832	.0455736	1.96	0.050	1.000085	1.178932
maxpreqtc	1.000907	.0003365	2.70	0.007	1.000248	1.001567
race_caucasian	.9030415	.0617515	-1.49	0.136	.7897709	1.032558
race_black	.790722	.0788136	-2.36	0.018	.6504023	.9613148
ethnicity_hisp	.8489509	.0644656	-2.16	0.031	.7315534	.9851879
1.icu	6.385464	.4521411	26.18	0.000	5.558028	7.336083
1.bpa_fired	1.422616	.1850352	2.71	0.007	1.10249	1.835696
icu#bpa_fired						
1 1	.920089	.1311797	-0.58	0.559	.6957795	1.216713
tele	.95361	.0757961	-0.60	0.550	.8160456	1.114364
ob	.4052396	.3128398	-1.17	0.242	.0892473	1.840046
stepdown	.9902698	.0406811	-0.24	0.812	.9136617	1.073301
cluster_4						
1	3.145475	.4633514	7.78	0.000	2.356666	4.198309
2	4.298126	.6408456	9.78	0.000	3.208972	5.756948
3	2.050313	.3079893	4.78	0.000	1.527412	2.752225
magnesium_time	1.000358	.0001136	3.15	0.002	1.000136	1.000581
magnesium_value	1.357215	.0614066	6.75	0.000	1.242043	1.483068
potassium_interval	.999316	.0003569	-1.92	0.055	.9986167	1.000016
potassium_value	1.185586	.0319401	6.32	0.000	1.124609	1.24987
creat_interval	1.000173	.0003652	0.48	0.635	.999458	1.00089
creatinine_value	1.044486	.0087763	5.18	0.000	1.027425	1.06183
1.admin_yn	.8893843	.032629	-3.20	0.001	.8276778	.9556912
med						
CITALOPRAM	.4136722	.0562746	-6.49	0.000	.3168559	.5400711
DROPERIDOL	.6021264	.0915163	-3.34	0.001	.4470076	.811074
ESCITALOPRAM	.468514	.0511387	-6.95	0.000	.3782799	.5802723
HALOPERIDOL	.8429255	.0511255	-2.82	0.005	.7484482	.9493288
HYDROXYCHLOROQUINE	.8064312	.1304516	-1.33	0.184	.5873183	1.107289
LEVOFLOXACIN	.7101573	.0513287	-4.74	0.000	.6163557	.8182343
METHADONE	.657653	.103059	-2.67	0.007	.4837348	.8941003
ONDANSETRON	.6703468	.0342221	-7.83	0.000	.6065192	.7408913
SOTALOL	.370084	.0923935	-3.98	0.000	.2268784	.603681
_cons	.0002867	.000077	-30.37	0.000	.0001694	.0004853

Note: **_cons** estimates baseline odds (conditional on zero random effects).

Supplemental Table 5C. Multivariable marginal logistic regression model for inpatient mortality in adjusted models with telemetry-alert interaction. bpa_fired = alert fired after medication order; admin_yn = medication was administered irrespective of alert firing.

```
GEE population-averaged model                   Number of obs    =  63,858
Group variable: arb_personid                    Number of groups =  39,381
Family: Binomial                                Obs per group:
Link:   Logit                                                min =       1
Correlation: exchangeable                                    avg =     1.6
                                                             max =      31
                                                Wald chi2(31)    = 2918.86
Scale parameter = 1                             Prob > chi2      =  0.0000
```

inpatient_mortality	Odds ratio	Std. err.	z	P>|z|	[95% conf.	interval]
age	1.031567	.0015091	21.24	0.000	1.028614	1.034529
female	1.085658	.0455657	1.96	0.050	.9999257	1.178741
maxpreqtc	1.000907	.0003364	2.70	0.007	1.000248	1.001567
race_caucasian	.9028437	.0617414	−1.49	0.135	.789592	1.032339
race_black	.790795	.0788134	−2.36	0.019	.6504743	.9613857
ethnicity_hisp	.8490573	.0644756	−2.15	0.031	.7316418	.9853158
1.icu	6.323351	.4364803	26.72	0.000	5.523211	7.239406
1.tele	.9711715	.078642	−0.36	0.718	.8286454	1.138212
1.bpa_fired	1.571806	.2653738	2.68	0.007	1.128984	2.188318
tele#bpa_fired						
1 1	.8302545	.1474101	−1.05	0.295	.5862488	1.175819
ob	.4057451	.3133195	−1.17	0.243	.08932	1.843137
stepdown	.9905395	.0406871	−0.23	0.817	.9139199	1.073583
cluster_4						
1	3.14449	.4631232	7.78	0.000	2.35605	4.196777
2	4.297888	.6407058	9.78	0.000	3.208948	5.756355
3	2.049529	.3078185	4.78	0.000	1.526905	2.751033
magnesium_time	1.000359	.0001136	3.16	0.002	1.000136	1.000581
magnesium_value	1.356949	.0613962	6.75	0.000	1.241796	1.482781
potassium_interval	.9993154	.0003571	−1.92	0.055	.9986158	1.000015
potassium_value	1.185594	.0319357	6.32	0.000	1.124624	1.249868
creat_interval	1.000174	.0003653	0.48	0.633	.9994587	1.000891
creatinine_value	1.044461	.008775	5.18	0.000	1.027403	1.061802
1.admin_yn	.8896138	.0326267	−3.19	0.001	.8279109	.9559153
med						
CITALOPRAM	.4141036	.0562871	−6.49	0.000	.3172555	.5405162
DROPERIDOL	.6018113	.0914466	−3.34	0.001	.4468053	.8105921
ESCITALOPRAM	.4690545	.0511719	−6.94	0.000	.3787571	.5808792
HALOPERIDOL	.8431635	.0511269	−2.81	0.005	.7486822	.949568
HYDROXYCHLOROQUINE	.8068372	.1305063	−1.33	0.185	.5876296	1.107817
LEVOFLOXACIN	.7105578	.0513479	−4.73	0.000	.6167201	.8186736
METHADONE	.6579376	.1030664	−2.67	0.008	.4839978	.8943881
ONDANSETRON	.6706663	.0342305	−7.83	0.000	.6068223	.7412275
SOTALOL	.3688012	.0919015	−4.00	0.000	.2262984	.6010395
_cons	.0002846	.0000765	−30.38	0.000	.0001681	.0004819

Note: _cons estimates baseline odds (conditional on zero random effects).

Supplemental Table 5D. Multivariable marginal logistic regression model for inpatient mortality with medication-alert interaction. Note that omnibus P value across medications (degrees of freedom = 9) had P value of 0.297 (chi-square 10.70). bpa_fired = alert fired after medication order; admin_yn = medication was administered irrespective of alert firing.

```
GEE population-averaged model                  Number of obs    =  63,858
Group variable: arb_personid                   Number of groups =  39,381
Family: Binomial                               Obs per group:
Link:   Logit                                               min =       1
Correlation: exchangeable                                   avg =     1.6
                                                            max =      31
                                               Wald chi2(39)    = 2935.27
Scale parameter = 1                            Prob > chi2      =  0.0000
```

inpatient_mortality	Odds ratio	Std. err.	z	P>|z|	[95% conf.	interval]
age	1.031518	.0015095	21.21	0.000	1.028564	1.034481
female	1.088605	.0457205	2.02	0.043	1.002583	1.182007
maxpreqtc	1.000904	.0003367	2.69	0.007	1.000245	1.001564
race_caucasian	.9044319	.0618776	-1.47	0.142	.7909337	1.034217
race_black	.7922681	.0789904	-2.34	0.020	.6516373	.9632485
ethnicity_hisp	.8506267	.0646048	-2.13	0.033	.7329774	.9871598
1.icu	6.308393	.4354508	26.68	0.000	5.51014	7.222288
1.tele	.9575714	.0760089	-0.55	0.585	.8196068	1.11876
ob	.4032745	.3112646	-1.18	0.239	.0888397	1.830605
stepdown	.9895658	.0406764	-0.26	0.799	.9129684	1.07259
cluster_4						
1	3.139494	.4629358	7.76	0.000	2.351502	4.191544
2	4.287888	.640015	9.75	0.000	3.200311	5.745063
3	2.049597	.3081595	4.77	0.000	1.526473	2.751995
magnesium_time	1.000357	.0001136	3.14	0.002	1.000134	1.00058
magnesium_value	1.358359	.0614579	6.77	0.000	1.24309	1.484316
potassium_interval	.9993261	.0003581	-1.88	0.060	.9986246	1.000028
potassium_value	1.185759	.031944	6.32	0.000	1.124775	1.250051
creat_interval	1.000171	.0003662	0.47	0.641	.9994534	1.000889
creatinine_value	1.044503	.0087818	5.18	0.000	1.027432	1.061857
1.admin_yn	.8848963	.0325705	-3.32	0.001	.8233074	.9510923
med						
CITALOPRAM	.4369395	.0627781	-5.76	0.000	.3297034	.5790542
DROPERIDOL	.5725469	.0911925	-3.50	0.000	.419021	.7823234
ESCITALOPRAM	.4411359	.0531938	-6.79	0.000	.3482823	.5587447
HALOPERIDOL	.8102173	.0515482	-3.31	0.001	.7152301	.9178193
HYDROXYCHLOROQUINE	.8149341	.1373148	-1.21	0.225	.585729	1.133831
LEVOFLOXACIN	.676858	.0516234	-5.12	0.000	.5828773	.7859917
METHADONE	.6900655	.1150529	-2.23	0.026	.4977046	.9567731
ONDANSETRON	.6593732	.0349595	-7.85	0.000	.5942939	.7315792
SOTALOL	.5139623	.2360784	-1.45	0.147	.2089049	1.264485
1.bpa_fired	1.016433	.1827606	0.09	0.928	.7145416	1.445872
med#bpa_fired						
CITALOPRAM#1	.8221343	.3461656	-0.47	0.642	.3601955	1.876495
DROPERIDOL#1	1.855468	.9883743	1.16	0.246	.6531811	5.270759
ESCITALOPRAM#1	1.579173	.476149	1.52	0.130	.8745353	2.851558
HALOPERIDOL#1	1.518981	.3249949	1.95	0.051	.9986951	2.310319
HYDROXYCHLOROQUINE#1	.870467	.5111008	-0.24	0.813	.2753996	2.751321
LEVOFLOXACIN#1	1.638194	.4023488	2.01	0.044	1.012292	2.651092
METHADONE#1	.8276112	.3898418	-0.40	0.688	.3287551	2.083436
ONDANSETRON#1	1.26159	.2476052	1.18	0.236	.8587324	1.853442
SOTALOL#1	.8478948	.4742218	-0.30	0.768	.2833157	2.537542
_cons	.0002957	.0000795	-30.24	0.000	.0001746	.0005007

Note: _cons estimates baseline odds (conditional on zero random effects).

Supplemental Table 6. Multivariable marginal logistic regression model for three-month mortality and alert without interaction terms. bpa_fired = alert fired after medication order; admin_yn = medication was administered irrespective of alert firing.

```
GEE population-averaged model                   Number of obs     =  63,858
Group variable: arb_personid                    Number of groups  =  39,381
Family: Binomial                                Obs per group:
Link:   Logit                                                 min =       1
Correlation: exchangeable                                     avg =     1.6
                                                              max =      31
                                                Wald chi2(30)     = 2746.34
Scale parameter = 1                             Prob > chi2       =  0.0000
```

threemo_mortality	Odds ratio	Std. err.	z	P>|z|	[95% conf.	interval]
age	1.031958	.0011526	28.16	0.000	1.029701	1.03422
female	.9953386	.0323078	-0.14	0.886	.9339886	1.060718
maxpreqtc	1.001681	.0002562	6.57	0.000	1.001179	1.002184
race_caucasian	1.025989	.0565272	0.47	0.641	.9209702	1.142984
race_black	.8392683	.0665058	-2.21	0.027	.7185375	.9802847
ethnicity_hisp	.9089096	.0540862	-1.61	0.108	.8088509	1.021346
icu	2.004259	.0771756	18.06	0.000	1.858564	2.161374
tele	1.186491	.0476962	4.25	0.000	1.096596	1.283755
ob	.2639199	.1955302	-1.80	0.072	.0617793	1.127461
stepdown	1.096967	.0342307	2.97	0.003	1.031887	1.166153
cluster_4						
1	2.114967	.18774	8.44	0.000	1.777235	2.51688
2	2.643009	.2429675	10.57	0.000	2.207238	3.164814
3	1.552636	.1398621	4.88	0.000	1.301347	1.852449
magnesium_time	1.000599	.0000963	6.22	0.000	1.00041	1.000788
magnesium_value	1.190073	.0423502	4.89	0.000	1.109896	1.276041
potassium_interval	1.000073	.0003282	0.22	0.823	.9994305	1.000717
potassium_value	1.098897	.0225307	4.60	0.000	1.055613	1.143955
creat_interval	.9997377	.0003348	-0.78	0.434	.9990817	1.000394
creatinine_value	1.040945	.0072726	5.74	0.000	1.026788	1.055297
1.admin_yn	.9108244	.0232297	-3.66	0.000	.8664143	.9575109
1.bpa_fired	1.303828	.0554234	6.24	0.000	1.199602	1.417109
med						
CITALOPRAM	.6384678	.0527197	-5.43	0.000	.543067	.7506278
DROPERIDOL	.7111717	.0668831	-3.62	0.000	.5914556	.8551196
ESCITALOPRAM	.7199721	.0490542	-4.82	0.000	.6299706	.8228317
HALOPERIDOL	.8708301	.038978	-3.09	0.002	.7976898	.9506767
HYDROXYCHLOROQUINE	.8571472	.1007725	-1.31	0.190	.680741	1.079267
LEVOFLOXACIN	.888868	.0446094	-2.35	0.019	.8055976	.9807455
METHADONE	.7906862	.0869434	-2.14	0.033	.637392	.9808481
ONDANSETRON	.8025445	.029193	-6.05	0.000	.7473193	.8618507
SOTALOL	.3963305	.0631993	-5.80	0.000	.2899505	.5417401
_cons	.0014721	.0002804	-34.24	0.000	.0010134	.0021382

Note: **_cons** estimates baseline odds (conditional on zero random effects).

Supplemental Table 7. Multivariable marginal logistic regression model for 6-month mortality and alert without interaction terms. bpa_fired = alert fired after medication order; admin_yn = medication was administered irrespective of alert firing.

```
GEE population-averaged model                  Number of obs    =  63,858
Group variable: arb_personid                   Number of groups =  39,381
Family: Binomial                               Obs per group:
Link:   Logit                                               min =       1
Correlation: exchangeable                                   avg =     1.6
                                                            max =      31
                                               Wald chi2(30)    = 2553.37
Scale parameter = 1                            Prob > chi2      =  0.0000
```

sixmo_mortality	Odds ratio	Std. err.	z	P>|z|	[95% conf.	interval]
age	1.031172	.0010838	29.21	0.000	1.02905	1.033299
female	.9909179	.0305512	-0.30	0.767	.932812	1.052643
maxpreqtc	1.002377	.0002347	10.14	0.000	1.001917	1.002837
race_caucasian	.9967433	.0522676	-0.06	0.950	.8993892	1.104635
race_black	.8166308	.0613387	-2.70	0.007	.7048398	.9461524
ethnicity_hisp	.8806163	.0500533	-2.24	0.025	.7877807	.9843921
icu	1.615082	.0540878	14.31	0.000	1.512476	1.724649
tele	1.188445	.0397534	5.16	0.000	1.113029	1.268971
ob	.2465181	.1721452	-2.01	0.045	.0627263	.9688315
stepdown	1.12417	.0314945	4.18	0.000	1.064107	1.187625
cluster_4						
1	1.730965	.1287638	7.38	0.000	1.496127	2.002662
2	2.162795	.1683957	9.91	0.000	1.856695	2.519359
3	1.360411	.1023478	4.09	0.000	1.173902	1.576553
magnesium_time	1.00044	.0000914	4.82	0.000	1.000261	1.00062
magnesium_value	1.15209	.0371637	4.39	0.000	1.081506	1.227282
potassium_interval	.9999531	.0003197	-0.15	0.883	.9993267	1.00058
potassium_value	1.093003	.0198855	4.89	0.000	1.054715	1.132681
creat_interval	.9999054	.0003252	-0.29	0.771	.9992683	1.000543
creatinine_value	1.037661	.0064577	5.94	0.000	1.025081	1.050395
1.admin_yn	.9328325	.0200642	-3.23	0.001	.8943248	.9729983
1.bpa_fired	1.221496	.0453696	5.39	0.000	1.135733	1.313736
med						
CITALOPRAM	.7102067	.0479964	-5.06	0.000	.6220993	.8107925
DROPERIDOL	.7891571	.0583645	-3.20	0.001	.6826691	.9122559
ESCITALOPRAM	.7855421	.0442528	-4.28	0.000	.703425	.8772455
HALOPERIDOL	.8652064	.0331605	-3.78	0.000	.8025942	.9327032
HYDROXYCHLOROQUINE	.8738205	.0868431	-1.36	0.175	.7191625	1.061738
LEVOFLOXACIN	.9048809	.0385286	-2.35	0.019	.8324314	.9836359
METHADONE	.898412	.0804553	-1.20	0.232	.753786	1.070787
ONDANSETRON	.827016	.0255011	-6.16	0.000	.7785151	.8785384
SOTALOL	.4809551	.0607136	-5.80	0.000	.375537	.6159653
_cons	.0019947	.0003412	-36.35	0.000	.0014265	.0027892

Note: **_cons** estimates baseline odds (conditional on zero random effects).

Supplemental Table 8. Multivariable marginal logistic regression model for 1-year mortality and alert firing without interaction terms. bpa_fired = alert fired after medication order; admin_yn = medication was administered irrespective of alert firing.

```
GEE population-averaged model                   Number of obs    =  63,858
Group variable: arb_personid                    Number of groups =  39,381
Family: Binomial                                Obs per group:
Link:   Logit                                                min =       1
Correlation: exchangeable                                    avg =     1.6
                                                             max =      31
                                                Wald chi2(30)    = 2544.37
Scale parameter = 1                             Prob > chi2      =  0.0000
```

oneyr_mortality	Odds ratio	Std. err.	z	P>|z|	[95% conf.	interval]
age	1.031344	.0010157	31.34	0.000	1.029355	1.033336
female	.9469634	.0276163	-1.87	0.062	.8943543	1.002667
maxpreqtc	1.003571	.0002022	17.69	0.000	1.003174	1.003967
race_caucasian	1.014901	.0508886	0.29	0.768	.9199058	1.119707
race_black	.8614999	.0610015	-2.11	0.035	.7498646	.9897546
ethnicity_hisp	.9099387	.0490034	-1.75	0.080	.8187889	1.011235
icu	1.299851	.0348661	9.78	0.000	1.23328	1.370015
tele	1.126302	.0283174	4.73	0.000	1.072146	1.183193
ob	.3133081	.1702032	-2.14	0.033	.1080339	.9086216
stepdown	1.12674	.0258509	5.20	0.000	1.077196	1.178564
cluster_4						
1	1.342169	.0751688	5.25	0.000	1.202639	1.497888
2	1.501556	.0898549	6.79	0.000	1.335379	1.688412
3	1.109528	.0626395	1.84	0.066	.9933053	1.239349
magnesium_time	1.00046	.0000795	5.78	0.000	1.000304	1.000616
magnesium_value	1.065988	.0287897	2.37	0.018	1.011029	1.123935
potassium_interval	.9999084	.0002915	-0.31	0.753	.9993373	1.00048
potassium_value	1.088704	.0159022	5.82	0.000	1.057979	1.120323
creat_interval	1.000021	.0002954	0.07	0.944	.9994422	1.0006
creatinine_value	1.036429	.0052506	7.06	0.000	1.026189	1.046772
1.admin_yn	.9478579	.0151315	-3.35	0.001	.9186599	.9779839
1.bpa_fired	1.137223	.0327836	4.46	0.000	1.07475	1.203327
med						
CITALOPRAM	.7788219	.0385763	-5.05	0.000	.7067679	.8582218
DROPERIDOL	.9054376	.046284	-1.94	0.052	.8191188	1.000853
ESCITALOPRAM	.8442952	.0351777	-4.06	0.000	.7780883	.9161355
HALOPERIDOL	.9018241	.0261927	-3.56	0.000	.8519212	.9546502
HYDROXYCHLOROQUINE	.9911428	.0718297	-0.12	0.902	.8599006	1.142416
LEVOFLOXACIN	.9404822	.0301433	-1.91	0.056	.8832198	1.001457
METHADONE	.9541999	.0631735	-0.71	0.479	.8380791	1.08641
ONDANSETRON	.8892235	.0206975	-5.04	0.000	.8495685	.9307296
SOTALOL	.5821509	.052416	-6.01	0.000	.4879717	.6945069
_cons	.0023743	.0003463	-41.44	0.000	.001784	.0031599

Note: **_cons** estimates baseline odds (conditional on zero random effects).

Supplemental Table 9A. Multivariable marginal logistic regression model for provider actions on risk of drug-induced QT prolongation (diLQTS). Note, across medications, the P value for interaction with alert response was 0.7929 (chi-square 5.46, df=9). action_grp = compliance or non-compliance with alert recommendations. See text for details.

GEE population-averaged model	Number of obs = **4,783**
Group variable: **arb_personid**	Number of groups = **4,096**
Family: Binomial	Obs per group:
Link: Logit	min = **1**
Correlation: **exchangeable**	avg = **1.2**
	max = **11**
	Wald chi2(**38**) = **138.90**
Scale parameter = **1**	Prob > chi2 = **0.0000**

dilqts	Odds ratio	Std. err.	z	P>|z|	[95% conf.	interval]
age	1.000047	.0024229	0.02	0.984	.99531	1.004807
female	.9564386	.0730396	-0.58	0.560	.8234819	1.110862
maxpreqtc	1.003819	.0005656	6.77	0.000	1.002711	1.004928
race_caucasian	.9023233	.1186191	-0.78	0.434	.6973704	1.167511
race_black	.9307848	.1611849	-0.41	0.679	.6628968	1.306931
ethnicity_hisp	.9193646	.1268613	-0.61	0.542	.7015073	1.204879
icu	1.62457	.1621991	4.86	0.000	1.335837	1.975711
tele	.9051787	.0903431	-1.00	0.318	.7443523	1.100754
ob	.7053953	.8070223	-0.31	0.760	.07492	6.641522
stepdown	1.167256	.0927135	1.95	0.052	.9989793	1.363879
cluster_4						
1	.9906394	.2133012	-0.04	0.965	.6495876	1.510753
2	.913666	.2044991	-0.40	0.687	.5892089	1.41679
3	1.072101	.2287865	0.33	0.744	.7056498	1.628854
magnesium_time	.9997076	.0002625	-1.11	0.265	.9991932	1.000222
magnesium_value	1.33663	.125107	3.10	0.002	1.112602	1.605767
potassium_interval	.9986614	.0008611	-1.55	0.120	.9969752	1.00035
potassium_value	.909801	.0477584	-1.80	0.072	.8208506	1.00839
creat_interval	1.000869	.0008718	1.00	0.319	.9991619	1.002579
creatinine_value	1.001764	.0209499	0.08	0.933	.9615333	1.043678
med						
CITALOPRAM	.9516567	.2458091	-0.19	0.848	.5736123	1.578855
DROPERIDOL	.7538555	.2681422	-0.79	0.427	.3754226	1.513755
ESCITALOPRAM	1.227195	.2749291	0.91	0.361	.7910767	1.903745
HALOPERIDOL	1.087085	.2296144	0.40	0.693	.7185752	1.644578
HYDROXYCHLOROQUINE	1.22282	.4746147	0.52	0.604	.571458	2.616619
LEVOFLOXACIN	1.048648	.2665129	0.19	0.852	.6372322	1.725686
METHADONE	1.551325	.4407029	1.55	0.122	.8889804	2.707157
ONDANSETRON	1.082812	.2099676	0.41	0.682	.7404523	1.583468
SOTALOL	1.967448	.4193272	3.18	0.001	1.29564	2.987597
action_grp						
Comply	1.113444	.288516	0.41	0.678	.670046	1.850257
med#action_grp						
CITALOPRAM#Comply	1.52292	.7234086	0.89	0.376	.6002666	3.863758
DROPERIDOL#Comply	1.241162	.7211968	0.37	0.710	.3973971	3.876436
ESCITALOPRAM#Comply	.7458561	.3282976	-0.67	0.505	.3147664	1.767347
HALOPERIDOL#Comply	.8350198	.2937419	-0.51	0.608	.4190467	1.663915
HYDROXYCHLOROQUINE#Comply	1.540305	1.134471	0.59	0.558	.3636444	6.524341
LEVOFLOXACIN#Comply	1.014792	.3750366	0.04	0.968	.4918117	2.093898
METHADONE#Comply	.8738159	.4385043	-0.27	0.788	.3267866	2.336554
ONDANSETRON#Comply	.8665839	.2418056	-0.51	0.608	.5015303	1.497352
SOTALOL#Comply	1.407181	.566232	0.85	0.396	.6394948	3.096441
_cons	.0322221	.0153985	-7.19	0.000	.0126292	.0822113

Note: **_cons** estimates baseline odds (conditional on zero random effects).

Supplemental Table 9B. Multivariable marginal logistic regression model for provider actions on risk of inpatient mortality. Note, across medications, the P value for interaction with alert response was 0.1837 (chi-square 12.56, df=9). action_grp = compliance or non-compliance with alert recommendations. See text for details.

```
GEE population-averaged model                     Number of obs    =  4,778
Group variable: arb_personid                      Number of groups =  4,091
Family: Binomial                                  Obs per group:
Link:   Logit                                                min =      1
Correlation: exchangeable                                    avg =    1.2
                                                             max =     11
                                                  Wald chi2(37)    = 357.33
Scale parameter = 1                               Prob > chi2      = 0.0000
```

inpatient_mortality	Odds ratio	Std. err.	z	P>|z|	[95% conf. interval]	
age	1.023688	.0041488	5.78	0.000	1.015589	1.031852
female	1.090107	.1275895	0.74	0.461	.8666461	1.371186
maxpreqtc	1.001726	.0008553	2.02	0.043	1.000051	1.003404
race_caucasian	.7108027	.1338051	-1.81	0.070	.4914899	1.027977
race_black	.3971391	.1152189	-3.18	0.001	.2249003	.7012861
ethnicity_hisp	.6722355	.1409469	-1.89	0.058	.4457085	1.013893
icu	6.375876	1.214806	9.72	0.000	4.388933	9.26234
tele	.9581837	.2222681	-0.18	0.854	.608131	1.509734
ob	1	(omitted)				
stepdown	.7342251	.0826471	-2.74	0.006	.5888638	.9154689
cluster_4						
1	2.908643	1.415017	2.19	0.028	1.120965	7.547254
2	3.889218	1.904464	2.77	0.006	1.489525	10.15493
3	2.475068	1.210935	1.85	0.064	.9487106	6.457143
magnesium_time	.9997274	.0003238	-0.84	0.400	.9990929	1.000362
magnesium_value	1.11809	.1471565	0.85	0.396	.8638665	1.447128
potassium_interval	1.000391	.0011355	0.34	0.731	.998168	1.002619
potassium_value	1.322278	.0933129	3.96	0.000	1.151472	1.51842
creat_interval	.9988738	.0011328	-0.99	0.320	.996656	1.001097
creatinine_value	1.079118	.0307683	2.67	0.008	1.020467	1.141139
med						
CITALOPRAM	.1744032	.0989707	-3.08	0.002	.0573473	.5303909
DROPERIDOL	.7343378	.3931148	-0.58	0.564	.2571699	2.096871
ESCITALOPRAM	.776454	.231325	-0.85	0.396	.4330337	1.392226
HALOPERIDOL	1.582418	.3974856	1.83	0.068	.9671832	2.589008
HYDROXYCHLOROQUINE	.5129451	.3184397	-1.08	0.282	.1519286	1.731819
LEVOFLOXACIN	1.176153	.3430592	0.56	0.578	.6640273	2.08325
METHADONE	.6868444	.3108574	-0.83	0.407	.2828883	1.667638
ONDANSETRON	.9264522	.2263246	-0.31	0.754	.5739598	1.495425
SOTALOL	.4379773	.1545091	-2.34	0.019	.2193642	.8744551
action_grp						
Comply	1.027879	.3304079	0.09	0.932	.5474276	1.930001
med#action_grp						
CITALOPRAM#Comply	3.500832	2.808575	1.56	0.118	.7265858	16.86769
DROPERIDOL#Comply	3.574761	2.825712	1.61	0.107	.7592845	16.8302
ESCITALOPRAM#Comply	.8811206	.5252022	-0.21	0.832	.2739472	2.834027
HALOPERIDOL#Comply	.5050209	.2153067	-1.60	0.109	.2189851	1.164673
HYDROXYCHLOROQUINE#Comply	1.656605	1.691098	0.49	0.621	.2240239	12.25021
LEVOFLOXACIN#Comply	1.004678	.4293652	0.01	0.991	.4347625	2.321675
METHADONE#Comply	.7496442	.5904703	-0.37	0.714	.1601001	3.510095
ONDANSETRON#Comply	.7949125	.2798588	-0.65	0.514	.3986973	1.584876
SOTALOL#Comply	.8381152	.6890467	-0.21	0.830	.1673009	4.198645
_cons	.0004543	.0003715	-9.41	0.000	.0000915	.0022562

Note: **_cons** estimates baseline odds (conditional on zero random effects).

Supplemental Table 9C. Multivariable marginal logistic regression model for provider actions on risk of 3-month mortality. Note, across medications, the P value for interaction with alert response was 0.2531 (chi-square 11.34, df=9). action_grp = compliance or non-compliance with alert recommendations. See text for details.

```
GEE population-averaged model                   Number of obs    =  4,778
Group variable: arb_personid                    Number of groups =  4,091
Family: Binomial                                Obs per group:
Link:   Logit                                                min =      1
Correlation: exchangeable                                    avg =    1.2
                                                             max =     11
                                                Wald chi2(37)    = 338.28
Scale parameter = 1                             Prob > chi2      = 0.0000
```

threemo_mortality	Odds ratio	Std. err.	z	P>|z|	[95% conf.	interval]
age	1.026718	.0032402	8.35	0.000	1.020387	1.033088
female	1.083921	.0988577	0.88	0.377	.9064935	1.296076
maxpreqtc	1.001972	.0006728	2.93	0.003	1.000654	1.003291
race_caucasian	.9414995	.147244	-0.39	0.700	.6929439	1.279211
race_black	.6800606	.1505445	-1.74	0.082	.4406743	1.049488
ethnicity_hisp	.8611821	.1432076	-0.90	0.369	.6216516	1.193007
icu	2.233799	.261237	6.87	0.000	1.776226	2.809247
tele	1.115042	.1485541	0.82	0.414	.8587919	1.447753
ob	1	(omitted)				
stepdown	.9138685	.0828191	-0.99	0.320	.7651451	1.0915
cluster_4						
1	1.691249	.4935519	1.80	0.072	.9545621	2.996478
2	2.223245	.6616236	2.68	0.007	1.240724	3.983818
3	1.365346	.4006583	1.06	0.289	.7681737	2.426757
magnesium_time	1.000335	.000252	1.33	0.184	.9998412	1.000829
magnesium_value	1.06007	.1138837	0.54	0.587	.8587951	1.308517
potassium_interval	1.000013	.0009224	0.01	0.988	.9982071	1.001823
potassium_value	1.146999	.0666191	2.36	0.018	1.023585	1.285292
creat_interval	.9996916	.0009278	-0.33	0.740	.9978747	1.001512
creatinine_value	1.072039	.0244047	3.06	0.002	1.025258	1.120954
med						
CITALOPRAM	.6229932	.1764349	-1.67	0.095	.3576173	1.085296
DROPERIDOL	.6371707	.2662266	-1.08	0.281	.2809321	1.445141
ESCITALOPRAM	.7024685	.1691996	-1.47	0.143	.4381298	1.126292
HALOPERIDOL	1.335743	.2781311	1.39	0.164	.888145	2.008916
HYDROXYCHLOROQUINE	.4354473	.2311373	-1.57	0.117	.1538559	1.232415
LEVOFLOXACIN	1.201841	.2922901	0.76	0.450	.7461618	1.935802
METHADONE	1.071943	.3467223	0.21	0.830	.5686552	2.020667
ONDANSETRON	.9572696	.1886874	-0.22	0.825	.6505099	1.408687
SOTALOL	.3955954	.1072973	-3.42	0.001	.2324769	.6731668
action_grp						
Comply	.7600789	.2093681	-1.00	0.319	.4429864	1.304148
med#action_grp						
CITALOPRAM#Comply	1.498	.8088264	0.75	0.454	.5198954	4.316259
DROPERIDOL#Comply	1.709884	1.267404	0.72	0.469	.3999786	7.309646
ESCITALOPRAM#Comply	2.34243	1.042142	1.91	0.056	.9794198	5.602274
HALOPERIDOL#Comply	.8168952	.29704	-0.56	0.578	.4005455	1.666023
HYDROXYCHLOROQUINE#Comply	1.702631	1.599615	0.57	0.571	.2700352	10.73546
LEVOFLOXACIN#Comply	1.434598	.524553	0.99	0.324	.7006372	2.93743
METHADONE#Comply	.3973118	.2941345	-1.25	0.212	.0931059	1.695453
ONDANSETRON#Comply	1.10004	.3278888	0.32	0.749	.6133251	1.972995
SOTALOL#Comply	.6063933	.476837	-0.64	0.525	.1298412	2.832019
_cons	.0029016	.0017016	-9.96	0.000	.0009193	.0091582

Note: **_cons** estimates baseline odds (conditional on zero random effects).

Supplemental Table 9D. Multivariable marginal logistic regression model for provider actions on risk of 6-month mortality. Note, across medications, the P value for interaction with alert response was 0.3394 (chi-square 10.14, df=9). action_grp = compliance or non-compliance with alert recommendations. See text for details.

```
GEE population-averaged model                   Number of obs    =  4,778
Group variable: arb_personid                    Number of groups =  4,091
Family: Binomial                                Obs per group:
Link:   Logit                                                min =      1
Correlation: exchangeable                                    avg =    1.2
                                                             max =     11
                                                Wald chi2(37)    = 320.59
Scale parameter = 1                             Prob > chi2      = 0.0000
```

sixmo_mortality	Odds ratio	Std. err.	z	P>|z|	[95% conf.	interval]
age	1.026999	.0030778	8.89	0.000	1.020984	1.033049
female	1.070728	.0931445	0.79	0.432	.9028835	1.269775
maxpreqtc	1.002347	.0006374	3.69	0.000	1.001098	1.003597
race_caucasian	.9844569	.1480563	-0.10	0.917	.7331304	1.321941
race_black	.6788892	.1440782	-1.82	0.068	.4478694	1.029073
ethnicity_hisp	.905441	.1437329	-0.63	0.531	.6633419	1.235899
icu	1.941741	.2063473	6.24	0.000	1.576649	2.391376
tele	1.197828	.141638	1.53	0.127	.9500438	1.510238
ob	1	(omitted)				
stepdown	.8937961	.0766413	-1.31	0.190	.7555263	1.057371
cluster_4						
1	1.550029	.4068241	1.67	0.095	.9266833	2.592675
2	1.942723	.5222391	2.47	0.013	1.147077	3.290253
3	1.372444	.3609098	1.20	0.229	.8197006	2.297915
magnesium_time	1.000446	.0002428	1.84	0.066	.9999704	1.000922
magnesium_value	1.06278	.1078796	0.60	0.549	.8710443	1.29672
potassium_interval	.9987585	.0008362	-1.48	0.138	.9971209	1.000399
potassium_value	1.110323	.0611116	1.90	0.057	.9967806	1.236799
creat_interval	1.001003	.0008413	1.19	0.233	.9993555	1.002653
creatinine_value	1.065343	.0233004	2.89	0.004	1.02064	1.112004
med						
CITALOPRAM	.6774398	.1672542	-1.58	0.115	.4175574	1.09907
DROPERIDOL	.7757573	.2629921	-0.75	0.454	.3991693	1.507629
ESCITALOPRAM	.6820775	.1469306	-1.78	0.076	.4471683	1.040391
HALOPERIDOL	1.181685	.2257346	0.87	0.382	.8126405	1.718323
HYDROXYCHLOROQUINE	.41504	.1940572	-1.88	0.060	.1659969	1.03772
LEVOFLOXACIN	.9700127	.2191278	-0.13	0.893	.6230018	1.510308
METHADONE	.8218755	.2514266	-0.64	0.521	.4512405	1.496939
ONDANSETRON	.8947392	.1600887	-0.62	0.534	.6300828	1.27056
SOTALOL	.3871082	.0937482	-3.92	0.000	.2408197	.6222613
action_grp						
Comply	.8125846	.2004022	-0.84	0.400	.5011209	1.317634
med#action_grp						
CITALOPRAM#Comply	1.094068	.5423958	0.18	0.856	.4140492	2.890923
DROPERIDOL#Comply	1.188488	.7627141	0.27	0.788	.3378582	4.180756
ESCITALOPRAM#Comply	1.959444	.7938102	1.66	0.097	.885715	4.334827
HALOPERIDOL#Comply	.7804702	.258548	-0.75	0.454	.4077341	1.493948
HYDROXYCHLOROQUINE#Comply	1.520928	1.281615	0.50	0.619	.2916356	7.931891
LEVOFLOXACIN#Comply	1.4046	.4724619	1.01	0.312	.7265005	2.715621
METHADONE#Comply	.4794981	.3135993	-1.12	0.261	.1330723	1.727771
ONDANSETRON#Comply	1.033947	.2768604	0.12	0.901	.6117472	1.747531
SOTALOL#Comply	.5368028	.3725222	-0.90	0.370	.1377561	2.091792
_cons	.0036385	.0019845	-10.30	0.000	.0012493	.010597

Note: _cons estimates baseline odds (conditional on zero random effects).

Supplemental Table 9E. Multivariable marginal logistic regression model for provider actions on risk of 1-year mortality. Note, across medications, the P value for interaction with alert response was 0.2053 (chi-square 12.14, df=9). action_grp = compliance or non-compliance with alert recommendations. See text for details.

```
GEE population-averaged model                    Number of obs    =  4,778
Group variable: arb_personid                     Number of groups =  4,091
Family: Binomial                                 Obs per group:
Link:   Logit                                                 min =      1
Correlation: exchangeable                                     avg =    1.2
                                                              max =     11
                                                 Wald chi2(37)    = 299.75
Scale parameter = 1                              Prob > chi2      = 0.0000
```

oneyr_mortality	Odds ratio	Std. err.	z	P>|z|	[95% conf.	interval]
age	1.023646	.0028436	8.41	0.000	1.018088	1.029234
female	1.067547	.0875779	0.80	0.426	.908986	1.253766
maxpreqtc	1.002622	.0005876	4.47	0.000	1.00147	1.003774
race_caucasian	.9898641	.1413221	-0.07	0.943	.748256	1.309486
race_black	.7334114	.1451389	-1.57	0.117	.4976209	1.080928
ethnicity_hisp	.9466845	.1417246	-0.37	0.714	.7059519	1.269508
icu	1.653817	.1538198	5.41	0.000	1.378218	1.984526
tele	1.101487	.1088573	0.98	0.328	.9075218	1.336908
ob	1	(omitted)				
stepdown	.9242276	.071485	-1.02	0.308	.7942225	1.075513
cluster_4						
1	.9917259	.195662	-0.04	0.966	.673681	1.45992
2	1.166745	.2395436	0.75	0.453	.7802189	1.74476
3	.8353396	.1646384	-0.91	0.361	.5676732	1.229215
magnesium_time	1.000428	.0002345	1.82	0.068	.9999683	1.000888
magnesium_value	1.07014	.0985919	0.74	0.462	.8933457	1.281922
potassium_interval	.9988097	.0007715	-1.54	0.123	.9972987	1.000323
potassium_value	1.209607	.0598347	3.85	0.000	1.097838	1.332754
creat_interval	1.000816	.0007714	1.06	0.290	.999305	1.002329
creatinine_value	1.045944	.0213752	2.20	0.028	1.004878	1.088689
med						
CITALOPRAM	.7874186	.1613397	-1.17	0.243	.5269835	1.176561
DROPERIDOL	.8211588	.2207344	-0.73	0.464	.4848607	1.390712
ESCITALOPRAM	.6956529	.1261817	-2.00	0.045	.4875247	.9926328
HALOPERIDOL	1.081581	.1800748	0.47	0.638	.7804417	1.498916
HYDROXYCHLOROQUINE	.6071546	.2030115	-1.49	0.136	.3152732	1.169261
LEVOFLOXACIN	.9371644	.1820105	-0.33	0.738	.6404725	1.371296
METHADONE	.7435134	.1977331	-1.11	0.265	.4414857	1.252163
ONDANSETRON	.8868058	.1368534	-0.78	0.436	.6553444	1.200017
SOTALOL	.4349091	.0886678	-4.08	0.000	.2916476	.6485427
action_grp						
Comply	.843168	.1792862	-0.80	0.422	.5558015	1.279112
med#action_grp						
CITALOPRAM#Comply	1.287969	.5186202	0.63	0.530	.5850001	2.835663
DROPERIDOL#Comply	.6946094	.3917636	-0.65	0.518	.2299631	2.098085
ESCITALOPRAM#Comply	1.568168	.5487118	1.29	0.199	.7898631	3.113389
HALOPERIDOL#Comply	.8687504	.2459812	-0.50	0.619	.4987496	1.513239
HYDROXYCHLOROQUINE#Comply	3.550159	1.959715	2.30	0.022	1.203311	10.47412
LEVOFLOXACIN#Comply	1.463846	.4233567	1.32	0.188	.8304641	2.5803
METHADONE#Comply	1.000212	.4556956	0.00	1.000	.4095299	2.44286
ONDANSETRON#Comply	1.116584	.257069	0.48	0.632	.7110842	1.753323
SOTALOL#Comply	.7984185	.3851397	-0.47	0.641	.3101913	2.055094
_cons	.0060556	.0028963	-10.68	0.000	.0023717	.0154621

Note: **_cons** estimates baseline odds (conditional on zero random effects).

Supplemental Table 9F. Multivariable marginal Poisson regression model for provider actions on hospital duration. Note, across medications, the P value for interaction with alert response was <0.0001 (chi-square 205.09, df=9). IRR = incidence-rate ratio. action_grp = compliance or non-compliance with alert recommendations. See text for details.

```
GEE population-averaged model                   Number of obs    =     4,763
Group variable: arb_personid                    Number of groups =     4,078
Family: Poisson                                 Obs per group:
Link:   Log                                                  min =         1
Correlation: exchangeable                                    avg =       1.2
                                                             max =        11
                                                Wald chi2(38)    = 36736.55
Scale parameter = 1                             Prob > chi2      =    0.0000
```

hospital_duration	IRR	Std. err.	z	P>|z|	[95% conf.	interval]
age	.9976603	.0002941	-7.95	0.000	.9970839	.9982369
female	.9117267	.0083576	-10.08	0.000	.8954924	.9282552
maxpreqtc	.999899	.0000689	-1.47	0.143	.999764	1.000034
race_caucasian	.9501308	.0142083	-3.42	0.001	.9226872	.9783907
race_black	1.034422	.0208185	1.68	0.093	.9944126	1.076041
ethnicity_hisp	1.042496	.0162702	2.67	0.008	1.01109	1.074878
icu	1.590942	.0196875	37.52	0.000	1.552819	1.630001
tele	1.303414	.0197785	17.46	0.000	1.265219	1.342761
ob	1.628223	.169505	4.68	0.000	1.327701	1.996768
stepdown	1.383008	.0125399	35.76	0.000	1.358648	1.407806
cluster_4						
1	.8247722	.018804	-8.45	0.000	.7887283	.8624633
2	1.065896	.0245469	2.77	0.006	1.018854	1.115109
3	.5418984	.0127078	-26.13	0.000	.5175553	.5673864
magnesium_time	1.001078	.0000131	82.32	0.000	1.001052	1.001103
magnesium_value	1.038495	.0111505	3.52	0.000	1.016869	1.060581
potassium_interval	1.000018	.0000751	0.23	0.815	.9998704	1.000165
potassium_value	1.008656	.0061016	1.42	0.154	.9967674	1.020686
creat_interval	1.000593	.0000754	7.87	0.000	1.000445	1.000741
creatinine_value	1.003496	.0025439	1.38	0.169	.9985222	1.008494
med						
CITALOPRAM	1.004247	.0311677	0.14	0.891	.9449808	1.067231
DROPERIDOL	1.023105	.0437925	0.53	0.594	.9407752	1.11264
ESCITALOPRAM	.9450676	.0253957	-2.10	0.036	.896581	.9961762
HALOPERIDOL	1.148992	.026598	6.00	0.000	1.098026	1.202324
HYDROXYCHLOROQUINE	.970745	.0444481	-0.65	0.517	.8874229	1.06189
LEVOFLOXACIN	1.03918	.0279573	1.43	0.153	.9858041	1.095446
METHADONE	.9060429	.0332601	-2.69	0.007	.8431442	.9736339
ONDANSETRON	1.033052	.0229473	1.46	0.143	.9890414	1.079021
SOTALOL	.6665303	.0203566	-13.28	0.000	.6278027	.7076468
action_grp						
Comply	.8907117	.0270742	-3.81	0.000	.8391971	.9453887
med#action_grp						
CITALOPRAM#Comply	1.43741	.0760832	6.86	0.000	1.295764	1.59454
DROPERIDOL#Comply	.6885486	.059034	-4.35	0.000	.5820432	.8145429
ESCITALOPRAM#Comply	1.47073	.0680714	8.33	0.000	1.343185	1.610387
HALOPERIDOL#Comply	1.249245	.0466081	5.96	0.000	1.161154	1.344018
HYDROXYCHLOROQUINE#Comply	1.365847	.1056716	4.03	0.000	1.173673	1.589487
LEVOFLOXACIN#Comply	1.503716	.0584963	10.49	0.000	1.393327	1.622851
METHADONE#Comply	1.290213	.066014	4.98	0.000	1.167104	1.426308
ONDANSETRON#Comply	1.310551	.0424516	8.35	0.000	1.229934	1.396453
SOTALOL#Comply	1.074915	.0732875	1.06	0.289	.9404582	1.228596
_cons	7.970779	.4395709	37.64	0.000	7.154164	8.880607

Note: **_cons** estimates baseline incidence rate (conditional on zero random effects).

Supplemental Table 10A. Multivariable marginal logistic regression model for impact of clinical decision-support (CDS) system on drug-induced QT prolongation (diLQTS) in medication-adjusted models. bpa_eligible = CDS system was enabled at the time of medication order.

```
GEE population-averaged model                   Number of obs     =  63,858
Group variable: arb_personid                    Number of groups  =  39,381
Family: Binomial                                Obs per group:
Link:   Logit                                                 min =       1
Correlation: exchangeable                                     avg =     1.6
                                                              max =      31
                                                Wald chi2(29)     = 1172.17
Scale parameter = 1                             Prob > chi2       =  0.0000
```

dilqts	Odds ratio	Std. err.	z	P>|z|	[95% conf.	interval]
age	1.000953	.0009721	0.98	0.327	.9990498	1.002861
female	1.002192	.0319368	0.07	0.945	.9415121	1.066783
maxpreqtc	1.002098	.0002507	8.38	0.000	1.001606	1.002589
race_caucasian	.9680007	.050325	−0.63	0.532	.8742245	1.071836
race_black	.9860592	.0704746	−0.20	0.844	.8571697	1.134329
ethnicity_hisp	.9554627	.0523818	−0.83	0.406	.8581197	1.063848
icu	1.80225	.0723611	14.67	0.000	1.665862	1.949805
tele	1.155893	.0461688	3.63	0.000	1.068855	1.250019
ob	.4849293	.1579648	−2.22	0.026	.2560951	.9182389
stepdown	1.250677	.0407214	6.87	0.000	1.173358	1.333091
cluster_4						
1	1.072498	.0836475	0.90	0.370	.9204678	1.249639
2	1.216895	.099638	2.40	0.017	1.036472	1.428725
3	1.246229	.0968811	2.83	0.005	1.070104	1.451342
magnesium_time	1.000034	.0001139	0.30	0.767	.9998106	1.000257
magnesium_value	1.348743	.0461871	8.74	0.000	1.261189	1.442375
potassium_interval	.999594	.0003627	−1.12	0.263	.9988833	1.000305
potassium_value	.7841901	.0177257	−10.75	0.000	.7502066	.8197129
creat_interval	1.000399	.00037	1.08	0.280	.9996745	1.001125
creatinine_value	1.011555	.0082003	1.42	0.156	.9956097	1.027756
1.bpa_eligible	1.12702	.1063226	1.27	0.205	.9367628	1.355919
med						
CITALOPRAM	1.299016	.1127416	3.01	0.003	1.095819	1.539892
DROPERIDOL	1.148255	.0978578	1.62	0.105	.9716202	1.357002
ESCITALOPRAM	1.163727	.0892613	1.98	0.048	1.001293	1.352511
HALOPERIDOL	1.065913	.0577066	1.18	0.238	.9586041	1.185235
HYDROXYCHLOROQUINE	1.369758	.1700648	2.53	0.011	1.073894	1.747135
LEVOFLOXACIN	.8872371	.0578492	−1.83	0.067	.7808005	1.008183
METHADONE	1.484301	.1559934	3.76	0.000	1.207993	1.82381
ONDANSETRON	1.124994	.0511833	2.59	0.010	1.029019	1.22992
SOTALOL	3.675479	.3817651	12.53	0.000	2.998481	4.505331
_cons	.0271997	.0054002	−18.16	0.000	.0184318	.0401385

Note: **_cons** estimates baseline odds (conditional on zero random effects).

Supplemental Table 10B. Multivariable marginal logistic regression model for impact of clinical decision-support (CDS) system on drug-induced QT prolongation (diLQTS) with medication interaction. Note that P value for interaction across all meds (df = 9) was 0.9754 (chi-square 2.69), P value for alert independent of meds was 0.1326 (chi-square 2.26, df = 1). bpa_eligible = CDS system was enabled at the time of medication order.

```
GEE population-averaged model                   Number of obs    =  63,858
Group variable: arb_personid                    Number of groups =  39,381
Family: Binomial                                Obs per group:
Link:   Logit                                                min =       1
Correlation: exchangeable                                    avg =     1.6
                                                             max =      31
                                                Wald chi2(38)    = 1176.22
Scale parameter = 1                             Prob > chi2      =  0.0000
```

dilqts	Odds ratio	Std. err.	z	P>|z|	[95% conf.	interval]
age	1.000943	.0009723	0.97	0.332	.9990395	1.002851
female	1.002238	.0319382	0.07	0.944	.9415552	1.066832
maxpreqtc	1.002098	.0002508	8.37	0.000	1.001607	1.002589
race_caucasian	.9673138	.0502862	-0.64	0.523	.8736094	1.071069
race_black	.9854282	.0704282	-0.21	0.837	.8566234	1.133601
ethnicity_hisp	.9548526	.0523484	-0.84	0.399	.8575716	1.063169
icu	1.802342	.0723791	14.67	0.000	1.665921	1.949935
tele	1.155844	.046174	3.63	0.000	1.068797	1.249981
ob	.4851335	.1580167	-2.22	0.026	.256218	.9185712
stepdown	1.251193	.0407433	6.88	0.000	1.173832	1.333652
cluster_4						
1	1.072721	.0836753	0.90	0.368	.9206415	1.249922
2	1.217651	.0997163	2.40	0.016	1.037089	1.42965
3	1.246687	.096926	2.84	0.005	1.070481	1.451896
magnesium_time	1.000036	.0001138	0.31	0.754	.9998126	1.000259
magnesium_value	1.348159	.0461778	8.72	0.000	1.260623	1.441773
potassium_interval	.9995922	.0003623	-1.13	0.261	.9988823	1.000303
potassium_value	.7840514	.0177233	-10.76	0.000	.7500727	.8195693
creat_interval	1.0004	.0003695	1.08	0.279	.999676	1.001125
creatinine_value	1.011579	.0081989	1.42	0.155	.9956362	1.027777
1.bpa_eligible	1.064728	.2897989	0.23	0.818	.6245362	1.81518
med						
CITALOPRAM	1.172752	.5083151	0.37	0.713	.5014911	2.742516
DROPERIDOL	.847754	.5477342	-0.26	0.798	.2389506	3.007679
ESCITALOPRAM	1.356993	.7495238	0.55	0.580	.4596461	4.006192
HALOPERIDOL	.6668999	.6450603	-0.42	0.675	.1001692	4.440042
HYDROXYCHLOROQUINE	.9883381	.5940758	-0.02	0.984	.304271	3.210336
LEVOFLOXACIN	1.011025	.3357064	0.03	0.974	.5273807	1.938205
METHADONE	1.215843	.6788265	0.35	0.726	.4070393	3.631769
ONDANSETRON	1.061042	.3033831	0.21	0.836	.6058274	1.858301
SOTALOL	1.468932	1.432368	0.39	0.693	.217264	9.93152
bpa_eligible#med						
1#CITALOPRAM	1.111461	.4911045	0.24	0.811	.4675052	2.64242
1#DROPERIDOL	1.362388	.887897	0.47	0.635	.3797999	4.88705
1#ESCITALOPRAM	.8557137	.4769806	-0.28	0.780	.2869875	2.551491
1#HALOPERIDOL	1.601021	1.55087	0.49	0.627	.2398054	10.68895
1#HYDROXYCHLOROQUINE	1.406096	.8610609	0.56	0.578	.4234079	4.669509
1#LEVOFLOXACIN	.868847	.2941115	-0.42	0.678	.4475123	1.68687
1#METHADONE	1.228945	.698179	0.36	0.717	.4036028	3.74206
1#ONDANSETRON	1.061754	.3072933	0.21	0.836	.6021003	1.872315
1#SOTALOL	2.541659	2.491239	0.95	0.341	.3722214	17.35534
_cons	.0288156	.0091423	-11.18	0.000	.0154728	.0536645

Note: **_cons** estimates baseline odds (conditional on zero random effects).

Supplemental Table 11. Multivariable marginal logistic regression model for impact of clinical decision-support (CDS) system on inpatient mortality. bpa_eligible = CDS system was enabled at the time of medication order.

```
GEE population-averaged model                   Number of obs    =  63,877
Group variable: arb_personid                    Number of groups =  39,394
Family: Binomial                                Obs per group:
Link:   Logit                                                min =       1
Correlation: exchangeable                                    avg =     1.6
                                                             max =      31
                                                Wald chi2(28)    = 2887.56
Scale parameter = 1                             Prob > chi2      =  0.0000
```

inpatient_mortality	Odds ratio	Std. err.	z	P>|z|	[95% conf.	interval]
age	1.03228	.0014888	22.03	0.000	1.029366	1.035202
female	1.087951	.0456064	2.01	0.044	1.002137	1.181112
race_caucasian	.9095653	.0621833	-1.39	0.166	.7955008	1.039985
race_black	.8052547	.0800866	-2.18	0.029	.6626391	.9785645
ethnicity_hisp	.8537248	.0647986	-2.08	0.037	.7357171	.9906608
icu	6.457332	.4467516	26.96	0.000	5.638487	7.395093
tele	.9586372	.0762901	-0.53	0.596	.8201894	1.120455
ob	.3879065	.3000865	-1.22	0.221	.0851595	1.766937
stepdown	.9961186	.0408953	-0.09	0.925	.9191053	1.079585
cluster_4						
1	3.21731	.4737358	7.94	0.000	2.410777	4.293672
2	4.397383	.6551644	9.94	0.000	3.283781	5.888633
3	2.06381	.3097216	4.83	0.000	1.537898	2.769567
magnesium_time	1.000416	.0001131	3.68	0.000	1.000194	1.000638
magnesium_value	1.366662	.0617558	6.91	0.000	1.250828	1.493222
potassium_interval	.9993388	.0003556	-1.86	0.063	.998642	1.000036
potassium_value	1.177193	.0318178	6.04	0.000	1.116454	1.241236
creat_interval	1.00015	.0003641	0.41	0.681	.9994363	1.000864
creatinine_value	1.047375	.0087998	5.51	0.000	1.030269	1.064765
1.bpa_eligible	1.431285	.1985205	2.59	0.010	1.090596	1.878402
med						
CITALOPRAM	.4383511	.0596356	-6.06	0.000	.3357532	.5723004
DROPERIDOL	.6130352	.0935038	-3.21	0.001	.4546267	.8266388
ESCITALOPRAM	.4950977	.0538635	-6.46	0.000	.4000236	.6127681
HALOPERIDOL	.8959338	.0522676	-1.88	0.060	.7991309	1.004463
HYDROXYCHLOROQUINE	.8444073	.1364287	-1.05	0.295	.6152131	1.158987
LEVOFLOXACIN	.7543696	.0535293	-3.97	0.000	.6564229	.8669311
METHADONE	.7071532	.110639	-2.21	0.027	.5203999	.9609259
ONDANSETRON	.6926423	.0350005	-7.27	0.000	.6273303	.764754
SOTALOL	.4696439	.1168196	-3.04	0.002	.2884298	.7647108
_cons	.0002543	.0000692	-30.40	0.000	.0001491	.0004336

Note: _cons estimates baseline odds (conditional on zero random effects).

Supplemental Table 12. Multivariable marginal logistic regression model for impact of clinical decision-support (CDS) system on 3-month mortality. bpa_eligible = CDS system was enabled at the time of medication order.

```
GEE population-averaged model                   Number of obs    =  63,877
Group variable: arb_personid                    Number of groups =  39,394
Family: Binomial                                Obs per group:
Link:   Logit                                                min =       1
Correlation: exchangeable                                    avg =     1.6
                                                             max =      31
                                                Wald chi2(28)    = 2672.18
Scale parameter = 1                             Prob > chi2      =  0.0000
```

threemo_mortality	Odds ratio	Std. err.	z	P>|z|	[95% conf.	interval]
age	1.033351	.0011406	29.72	0.000	1.031118	1.035589
female	.9990432	.0324276	-0.03	0.976	.9374657	1.064666
race_caucasian	1.032379	.0569113	0.58	0.563	.9266492	1.150172
race_black	.8590673	.0680209	-1.92	0.055	.7355786	1.003287
ethnicity_hisp	.910971	.0542552	-1.57	0.117	.8106045	1.023764
icu	2.053216	.0793847	18.61	0.000	1.903374	2.214854
tele	1.188857	.0480622	4.28	0.000	1.098292	1.286889
ob	.2495701	.1859395	-1.86	0.062	.0579447	1.074907
stepdown	1.104335	.0345273	3.17	0.002	1.038695	1.174124
cluster_4						
1	2.164054	.1924963	8.68	0.000	1.817826	2.576226
2	2.704799	.2490276	10.81	0.000	2.258218	3.239695
3	1.571101	.1417547	5.01	0.000	1.316447	1.875016
magnesium_time	1.00067	.0000964	6.95	0.000	1.000481	1.000858
magnesium_value	1.196235	.0426985	5.02	0.000	1.115408	1.282919
potassium_interval	1.000102	.0003257	0.31	0.755	.9994634	1.00074
potassium_value	1.088524	.0225411	4.10	0.000	1.045229	1.133613
creat_interval	.9997141	.0003327	-0.86	0.390	.9990622	1.000366
creatinine_value	1.046048	.0076526	6.15	0.000	1.031157	1.061155
1.bpa_eligible	1.859627	.2122216	5.44	0.000	1.486916	2.325763
med						
CITALOPRAM	.6750302	.0559741	-4.74	0.000	.573774	.7941556
DROPERIDOL	.7271812	.0689532	-3.36	0.001	.6038506	.8757008
ESCITALOPRAM	.7581283	.0515754	-4.07	0.000	.6634918	.8662631
HALOPERIDOL	.9150381	.0395525	-2.05	0.040	.8407096	.9959381
HYDROXYCHLOROQUINE	.8989007	.1059149	-0.90	0.366	.713538	1.132417
LEVOFLOXACIN	.9329252	.0460918	-1.41	0.160	.846823	1.027782
METHADONE	.8488989	.0936815	-1.48	0.138	.6837862	1.053881
ONDANSETRON	.825723	.0298045	-5.31	0.000	.7693257	.8862548
SOTALOL	.4990222	.0794795	-4.36	0.000	.3652149	.6818536
_cons	.0013661	.000271	-33.24	0.000	.000926	.0020155

Note: **_cons** estimates baseline odds (conditional on zero random effects).

Supplemental Table 13. Multivariable marginal logistic regression model for impact of clinical decision-support (CDS) system on 6-month mortality. bpa_eligible = CDS system was enabled at the time of medication order.

```
GEE population-averaged model                   Number of obs     =  63,877
Group variable: arb_personid                    Number of groups  =  39,394
Family: Binomial                                Obs per group:
Link:   Logit                                                min =        1
Correlation: exchangeable                                    avg =      1.6
                                                             max =       31
                                                Wald chi2(28)     = 2437.53
Scale parameter = 1                             Prob > chi2       =  0.0000
```

sixmo_mortality	Odds ratio	Std. err.	z	P>|z|	[95% conf.	interval]
age	1.033066	.0010752	31.26	0.000	1.030961	1.035176
female	.9957183	.0307117	-0.14	0.889	.9373078	1.057769
race_caucasian	1.003127	.0526585	0.06	0.953	.9050498	1.111832
race_black	.8413382	.0631615	-2.30	0.021	.7262207	.9747036
ethnicity_hisp	.8816875	.0501977	-2.21	0.027	.7885925	.9857725
icu	1.647016	.0555618	14.79	0.000	1.541639	1.759597
tele	1.194834	.0403983	5.26	0.000	1.118221	1.276696
ob	.2286965	.1618612	-2.08	0.037	.0571238	.915592
stepdown	1.132474	.0318976	4.42	0.000	1.071651	1.19675
cluster_4						
1	1.76452	.1319758	7.59	0.000	1.523918	2.043109
2	2.209145	.1728097	10.13	0.000	1.895131	2.575189
3	1.373884	.103896	4.20	0.000	1.184624	1.593381
magnesium_time	1.000503	.0000915	5.50	0.000	1.000323	1.000682
magnesium_value	1.161848	.0377182	4.62	0.000	1.090224	1.238177
potassium_interval	.9999826	.0003181	-0.05	0.956	.9993593	1.000606
potassium_value	1.084647	.0199998	4.41	0.000	1.046148	1.124563
creat_interval	.9998859	.0003239	-0.35	0.725	.9992513	1.000521
creatinine_value	1.042974	.0068508	6.41	0.000	1.029633	1.056488
1.bpa_eligible	1.789387	.1833151	5.68	0.000	1.463869	2.187289
med						
CITALOPRAM	.7453322	.0507333	-4.32	0.000	.6522443	.8517057
DROPERIDOL	.8096083	.0606637	-2.82	0.005	.6990283	.9376811
ESCITALOPRAM	.8209802	.0463191	-3.50	0.000	.7350358	.9169737
HALOPERIDOL	.9015233	.0333867	-2.80	0.005	.838405	.9693934
HYDROXYCHLOROQUINE	.9080959	.0907938	-0.96	0.335	.7464941	1.104681
LEVOFLOXACIN	.9408611	.0394391	-1.45	0.146	.8666519	1.021425
METHADONE	.9554916	.0862007	-0.50	0.614	.8006353	1.1403
ONDANSETRON	.8478721	.0259587	-5.39	0.000	.7984904	.9003078
SOTALOL	.5824094	.0739405	-4.26	0.000	.4541121	.7469536
_cons	.0025025	.0004428	-33.86	0.000	.0017692	.0035399

Note: **_cons** estimates baseline odds (conditional on zero random effects).

Supplemental Table 14. Multivariable marginal logistic regression model for impact of clinical decision-support (CDS) system on 1-year mortality. bpa_eligible = CDS system was enabled at the time of medication order.

```
GEE population-averaged model                   Number of obs    =  63,877
Group variable: arb_personid                    Number of groups =  39,394
Family: Binomial                                Obs per group:
Link:   Logit                                                min =       1
Correlation: exchangeable                                    avg =     1.6
                                                             max =      31
                                                Wald chi2(28)    = 2293.93
Scale parameter = 1                             Prob > chi2      =  0.0000
```

oneyr_mortality	Odds ratio	Std. err.	z	P>|z|	[95% conf.	interval]
age	1.034899	.0010189	34.84	0.000	1.032904	1.036898
female	.9445341	.0276164	-1.95	0.051	.8919286	1.000242
race_caucasian	1.022647	.0515421	0.44	0.657	.926456	1.128826
race_black	.898171	.0638251	-1.51	0.131	.7813968	1.032396
ethnicity_hisp	.9139342	.0495105	-1.66	0.097	.8218695	1.016312
icu	1.298978	.034458	9.86	0.000	1.233167	1.368301
tele	1.143006	.0285035	5.36	0.000	1.088483	1.200259
ob	.2917313	.1606572	-2.24	0.025	.0991343	.8585033
stepdown	1.142094	.0259339	5.85	0.000	1.092379	1.194071
cluster_4						
1	1.328521	.0732031	5.16	0.000	1.192521	1.48003
2	1.479025	.0871525	6.64	0.000	1.317704	1.660095
3	1.090036	.0605412	1.55	0.121	.9776081	1.215394
magnesium_time	1.000498	.0000783	6.36	0.000	1.000344	1.000651
magnesium_value	1.079329	.0289832	2.84	0.004	1.023992	1.137657
potassium_interval	.9999319	.0002887	-0.24	0.813	.9993662	1.000498
potassium_value	1.083755	.0157783	5.52	0.000	1.053267	1.115125
creat_interval	1.00002	.0002926	0.07	0.945	.9994469	1.000594
creatinine_value	1.040143	.0054335	7.53	0.000	1.029548	1.050847
1.bpa_eligible	1.786488	.1528558	6.78	0.000	1.51067	2.112666
med						
CITALOPRAM	.8118961	.0392509	-4.31	0.000	.7384981	.8925889
DROPERIDOL	.9516286	.0477284	-0.99	0.323	.8625335	1.049927
ESCITALOPRAM	.8800809	.0355555	-3.16	0.002	.813081	.9526018
HALOPERIDOL	.9369743	.0253703	-2.40	0.016	.8885459	.9880422
HYDROXYCHLOROQUINE	1.034418	.0729103	0.48	0.631	.9009475	1.187661
LEVOFLOXACIN	.9668303	.0294411	-1.11	0.268	.9108151	1.026291
METHADONE	1.014463	.0656913	0.22	0.825	.893546	1.151743
ONDANSETRON	.9122289	.0203436	-4.12	0.000	.8732151	.9529858
SOTALOL	.6743689	.0598374	-4.44	0.000	.5667213	.8024638
_cons	.0045377	.0006698	-36.55	0.000	.0033978	.0060601

Note: **_cons** estimates baseline odds (conditional on zero random effects).

Supplemental Table 15. Multivariable marginal Poisson regression model for impact of clinical decision-support (CDS) system on hospital duration. bpa_eligible = CDS system was enabled at the time of medication order; IRR = incidence-rate ratio.

```
GEE population-averaged model                   Number of obs    =     60,368
Group variable: arb_personid                    Number of groups =     37,297
Family: Poisson                                 Obs per group:
Link:   Log                                                  min =          1
Correlation: exchangeable                                    avg =        1.6
                                                             max =         31
                                                Wald chi2(28)    = 338516.21
Scale parameter = 1                             Prob > chi2      =     0.0000
```

hospital_duration	IRR	Std. err.	z	P>|z|	[95% conf.	interval]
age	.9987307	.0000956	-13.28	0.000	.9985434	.998918
female	.889199	.0028516	-36.62	0.000	.8836275	.8948056
race_caucasian	.9355437	.0046931	-13.28	0.000	.9263905	.9447874
race_black	.9640291	.0066974	-5.27	0.000	.9509914	.9772456
ethnicity_hisp	.9819959	.0052087	-3.43	0.001	.97184	.992258
icu	1.582563	.0060513	120.05	0.000	1.570747	1.594468
tele	1.256135	.0050834	56.35	0.000	1.246211	1.266138
ob	1.149733	.0280112	5.73	0.000	1.096122	1.205965
stepdown	1.35825	.004053	102.61	0.000	1.350329	1.366217
cluster_4						
1	.988753	.0064597	-1.73	0.083	.9761729	1.001495
2	1.353056	.0091274	44.82	0.000	1.335284	1.371064
3	.603411	.0040972	-74.40	0.000	.5954339	.6114951
magnesium_time	1.001304	4.51e-06	289.18	0.000	1.001295	1.001313
magnesium_value	.9870946	.0034845	-3.68	0.000	.9802886	.9939478
potassium_interval	1.000132	.0000169	7.78	0.000	1.000099	1.000165
potassium_value	.968118	.0020278	-15.47	0.000	.9641518	.9721006
creat_interval	1.000721	.0000173	41.81	0.000	1.000687	1.000755
creatinine_value	1.008744	.000761	11.54	0.000	1.007254	1.010237
1.bpa_eligible	.9647827	.0084727	-4.08	0.000	.9483186	.9815325
med						
CITALOPRAM	1.003249	.0082547	0.39	0.693	.9872003	1.01956
DROPERIDOL	.8936569	.0076545	-13.13	0.000	.8787797	.9087861
ESCITALOPRAM	1.021958	.0067994	3.26	0.001	1.008718	1.035372
HALOPERIDOL	1.175476	.0052754	36.02	0.000	1.165181	1.185861
HYDROXYCHLOROQUINE	1.094026	.012719	7.73	0.000	1.069379	1.11924
LEVOFLOXACIN	1.137839	.0057014	25.77	0.000	1.126719	1.149068
METHADONE	1.17553	.0102767	18.50	0.000	1.15556	1.195846
ONDANSETRON	1.071432	.0042425	17.42	0.000	1.063149	1.079779
SOTALOL	.7648936	.0129362	-15.85	0.000	.7399548	.7906729
_cons	7.989302	.1322702	125.52	0.000	7.734218	8.252798

Note: **_cons** estimates baseline incidence rate (conditional on zero random effects).

Supplemental Table 16A. Multivariable marginal Poisson regression model for impact of clinical decision-support (CDS) system on hospital duration in patients with drug-induced QT prolongation (diLQTS). Note that P value for drug-induced QT prolongation (diLQTS) across all medications (degrees of freedom = 9) was < 0.0001 (chi-square 88.86).

```
GEE population-averaged model                    Number of obs     =   177,893
Group variable: arb_personid                     Number of groups =   102,760
Family: Poisson                                  Obs per group:
Link:   Log                                                   min =         1
Correlation: exchangeable                                     avg =       1.7
                                                              max =        52
                                                 Wald chi2(31)     = 659613.86
Scale parameter = 1                              Prob > chi2       =    0.0000
```

hospital_duration	IRR	Std. err.	z	P>|z|	[95% conf.	interval]
1.dilqts	1.154523	.0107894	15.38	0.000	1.133568	1.175865
med						
CITALOPRAM	1.028055	.0077722	3.66	0.000	1.012934	1.043402
DROPERIDOL	.7309929	.0061586	-37.19	0.000	.7190214	.7431636
ESCITALOPRAM	1.08856	.0068697	13.45	0.000	1.075179	1.102108
HALOPERIDOL	1.134141	.0044714	31.93	0.000	1.12541	1.142938
HYDROXYCHLOROQUINE	1.095167	.011974	8.31	0.000	1.071948	1.118889
LEVOFLOXACIN	1.221284	.0055954	43.63	0.000	1.210367	1.2323
METHADONE	1.26708	.0111085	27.00	0.000	1.245494	1.28904
ONDANSETRON	1.011456	.0035265	3.27	0.001	1.004568	1.018392
SOTALOL	.8567144	.0146655	-9.03	0.000	.8284475	.8859458
dilqts#med						
1#CITALOPRAM	1.024191	.0201792	1.21	0.225	.9853946	1.064515
1#DROPERIDOL	1.166974	.0290244	6.21	0.000	1.111452	1.225271
1#ESCITALOPRAM	.9493008	.0173006	-2.85	0.004	.9159907	.9838222
1#HALOPERIDOL	.9772587	.0109352	-2.06	0.040	.9560594	.998928
1#HYDROXYCHLOROQUINE	1.025254	.0282099	0.91	0.365	.9714282	1.082063
1#LEVOFLOXACIN	.9900571	.013722	-0.72	0.471	.9635244	1.01732
1#METHADONE	1.031246	.0218835	1.45	0.147	.9892343	1.075041
1#ONDANSETRON	.9902012	.0098603	-0.99	0.323	.9710627	1.009717
1#SOTALOL	.8760823	.0282724	-4.10	0.000	.8223854	.9332852
age	.9990605	.0000718	-13.08	0.000	.9989198	.9992013
female	.8906419	.0021339	-48.34	0.000	.8864693	.894834
race_caucasian	.8844338	.0033923	-32.02	0.000	.87781	.8911076
race_black	1.012923	.0054452	2.39	0.017	1.002306	1.023652
ethnicity_hisp	.9996531	.0040654	-0.09	0.932	.9917168	1.007653
icu	1.724952	.0050015	188.03	0.000	1.715177	1.734783
tele	1.522881	.004522	141.65	0.000	1.514044	1.53177
ob	1.652273	.0234482	35.38	0.000	1.606949	1.698876
stepdown	1.53782	.0035938	184.16	0.000	1.530793	1.54488
cluster_4						
1	1.124718	.0053751	24.59	0.000	1.114232	1.135303
2	1.657273	.0084631	98.93	0.000	1.640769	1.673944
3	.5300762	.0025934	-129.73	0.000	.5250174	.5351836
_cons	4.518697	.037048	183.96	0.000	4.446665	4.591897

Note: **_cons** estimates baseline incidence rate (conditional on zero random effects).

Supplemental Table 16B. Estimated hospital duration for specific medications with and without drug-induced QT prolongation (diLQTS). Estimates obtained from model in Supplemental Table 16A (marginal estimation).

Medication	Duration with diLQTS	Duration without diLQTS	P value
Azithromycin	5.95 ± 0.05 days	5.16 ± 0.017 days	< 0.0001
Citalopram	6.27 ± 0.10 days	5.30 ± 0.036 days	0.225
Droperidol	5.08 ± 0.11 days	3.77 ± 0.029 days	<0.0001
Escitalopram	6.15 ± 0.09 days	5.61 ± 0.031 days	0.0043
Haloperidol	6.60 ± 0.041 days	5.85 ± 0.014 days	0.0398
Hydroxychloroquine	6.69 ± 0.16 days	5.65 ± 0.059 days	0.3647
Levofloxacin	7.20 ± 0.072 days	6.30 ± 0.021 days	0.4709
Methadone	7.78 ± 0.14 days	6.53 ± 0.053 days	0.1471
Ondansetron	5.96 ± 0.023 days	5.22 ± 0.0081 days	0.3227
Sotalol	4.47 ± 0.12 days	4.42 ± 0.074 days	< 0.0001

Supplemental Table 17. Multivariable marginal logistic regression model for impact of clinical decision-support (CDS) system on inpatient mortality in patients with drug-induced QT prolongation (diLQTS). Note that the P value for the interaction of diLQTS across all medications (df = 9) was 0.093 (chi-square 14.93), and for diLQTS independently P = 0.8659, df = 1, chi-square = 0.03.

```
GEE population-averaged model                   Number of obs    = 178,097
Group variable: arb_personid                    Number of groups = 102,847
Family: Binomial                                Obs per group:
Link:   Logit                                                min =       1
Correlation: exchangeable                                    avg =     1.7
                                                             max =      52
                                                Wald chi2(31)    = 7454.80
Scale parameter = 1                             Prob > chi2      =  0.0000
```

inpatient_mortality	Odds ratio	Std. err.	z	P>|z|	[95% conf.	interval]
1.dilqts	1.114016	.1236522	0.97	0.331	.8962119	1.384752
med						
CITALOPRAM	.437258	.0534382	-6.77	0.000	.3441206	.5556034
DROPERIDOL	.4833086	.0730982	-4.81	0.000	.3593224	.650077
ESCITALOPRAM	.4663413	.0474617	-7.50	0.000	.3820087	.5692914
HALOPERIDOL	.8465893	.0433154	-3.25	0.001	.7658107	.9358886
HYDROXYCHLOROQUINE	.8123146	.1243012	-1.36	0.174	.6018281	1.096418
LEVOFLOXACIN	.686433	.0450081	-5.74	0.000	.6036518	.7805664
METHADONE	.6797362	.1038955	-2.53	0.012	.5037755	.9171573
ONDANSETRON	.640036	.0283407	-10.08	0.000	.5868313	.6980644
SOTALOL	.4419014	.1190791	-3.03	0.002	.2605867	.7493739
dilqts#med						
1#CITALOPRAM	1.331794	.3829561	1.00	0.319	.7580097	2.33991
1#DROPERIDOL	1.383126	.5345062	0.84	0.401	.6485102	2.949897
1#ESCITALOPRAM	1.031794	.2882013	0.11	0.911	.5968086	1.78382
1#HALOPERIDOL	1.006162	.1420665	0.04	0.965	.7629239	1.32695
1#HYDROXYCHLOROQUINE	.405639	.1999194	-1.83	0.067	.1543919	1.065749
1#LEVOFLOXACIN	1.292475	.2369682	1.40	0.162	.9023168	1.851336
1#METHADONE	.5652532	.2478165	-1.30	0.193	.2393658	1.334824
1#ONDANSETRON	.8704088	.1075502	-1.12	0.261	.6831977	1.10892
1#SOTALOL	.815624	.4120344	-0.40	0.687	.3030279	2.195318
age	1.032359	.0011148	29.49	0.000	1.030177	1.034547
female	1.003851	.0310073	0.12	0.901	.9448803	1.066501
race_caucasian	.8086984	.0414047	-4.15	0.000	.7314855	.8940616
race_black	.794052	.0601247	-3.05	0.002	.6845371	.9210877
ethnicity_hisp	.8689132	.049532	-2.46	0.014	.7770592	.971625
icu	8.420301	.4210978	42.60	0.000	7.634123	9.287441
tele	1.220055	.0680463	3.57	0.000	1.093718	1.360986
ob	.300791	.2037522	-1.77	0.076	.0797391	1.134641
stepdown	1.067234	.033751	2.06	0.040	1.003091	1.135478
cluster_4						
1	3.136278	.3110539	11.52	0.000	2.582218	3.80922
2	4.249247	.43232	14.22	0.000	3.481052	5.186968
3	1.684658	.1692554	5.19	0.000	1.383543	2.051308
_cons	.0011121	.0001571	-48.15	0.000	.0008432	.0014668

Note: **_cons** estimates baseline odds (conditional on zero random effects).

Supplemental Table 18. Multivariable marginal logistic regression model for impact of clinical decision-support (CDS) system on 3-month mortality in patients with drug-induced QT prolongation (diLQTS). Note that the P value for interaction of diLQTS across all medications (df = 9) was 0.0318 (chi-square 18.3), and for diLQTS independently P = 0.5549, df = 1, chi-square = 0.35.

```
GEE population-averaged model                   Number of obs    = 178,097
Group variable: arb_personid                    Number of groups = 102,847
Family: Binomial                                Obs per group:
Link:   Logit                                                min =       1
Correlation: exchangeable                                    avg =     1.7
                                                             max =      52
                                                Wald chi2(31)    = 7174.01
Scale parameter = 1                             Prob > chi2      =  0.0000
```

threemo_mortality	Odds ratio	Std. err.	z	P>|z|	[95% conf.	interval]
1.dilqts	1.142828	.0976692	1.56	0.118	.9665735	1.351223
med						
CITALOPRAM	.7138898	.0500971	-4.80	0.000	.6221544	.8191513
DROPERIDOL	.636497	.0526197	-5.46	0.000	.5412861	.7484551
ESCITALOPRAM	.7493992	.0454794	-4.75	0.000	.6653584	.8440551
HALOPERIDOL	.825372	.0296201	-5.35	0.000	.7693123	.8855168
HYDROXYCHLOROQUINE	.951792	.1004451	-0.47	0.640	.7739493	1.1705
LEVOFLOXACIN	.9437595	.0403358	-1.35	0.176	.8679234	1.026222
METHADONE	.8891747	.0925782	-1.13	0.259	.725041	1.090465
ONDANSETRON	.781148	.0233286	-8.27	0.000	.7367373	.8282359
SOTALOL	.5770696	.0888491	-3.57	0.000	.4267486	.7803409
dilqts#med						
1#CITALOPRAM	.8732652	.1727249	-0.69	0.493	.5926322	1.286788
1#DROPERIDOL	1.088053	.2816319	0.33	0.744	.6551262	1.807072
1#ESCITALOPRAM	.9892523	.1749561	-0.06	0.951	.6994653	1.399097
1#HALOPERIDOL	1.036805	.1112083	0.34	0.736	.8402268	1.279373
1#HYDROXYCHLOROQUINE	.4677477	.160348	-2.22	0.027	.2388978	.9158219
1#LEVOFLOXACIN	.9453559	.131885	-0.40	0.687	.7191933	1.242639
1#METHADONE	.6638122	.1960694	-1.39	0.165	.3720719	1.184305
1#ONDANSETRON	.8226196	.0759424	-2.12	0.034	.6864644	.9857802
1#SOTALOL	.753943	.2325495	-0.92	0.360	.4118984	1.380025
age	1.036708	.000844	44.28	0.000	1.035055	1.038363
female	.910304	.0211344	-4.05	0.000	.8698097	.9526835
race_caucasian	.9389819	.0384124	-1.54	0.124	.866634	1.017369
race_black	.8717457	.0520599	-2.30	0.022	.7754554	.9799927
ethnicity_hisp	.8995901	.0399936	-2.38	0.017	.8245222	.9814926
icu	2.480923	.0693281	32.52	0.000	2.348696	2.620593
tele	1.336997	.0360403	10.77	0.000	1.268193	1.409534
ob	.2638866	.1327932	-2.65	0.008	.0984181	.7075538
stepdown	1.201862	.0284575	7.77	0.000	1.147361	1.258952
cluster_4						
1	2.02582	.1158921	12.34	0.000	1.810947	2.266188
2	2.732653	.1687171	16.28	0.000	2.421198	3.084172
3	1.319613	.075422	4.85	0.000	1.179768	1.476035
_cons	.0038447	.0003597	-59.45	0.000	.0032006	.0046184

Note: **_cons** estimates baseline odds (conditional on zero random effects).

Supplemental Table 19. Multivariable marginal logistic regression model for impact of clinical decision-support (CDS) system on 6-month mortality in patients with drug-induced QT prolongation (diLQTS). Note that the P value for interaction of diLQTS across all medications (df = 9) was 0.0256 (chi-square 18.95), and for diLQTS independently P = 0.6169, df = 1, chi-square = 0.25.

```
GEE population-averaged model                   Number of obs    = 178,097
Group variable: arb_personid                    Number of groups = 102,847
Family: Binomial                                Obs per group:
Link:   Logit                                                min =       1
Correlation: exchangeable                                    avg =     1.7
                                                             max =      52
                                                Wald chi2(31)    = 6743.28
Scale parameter = 1                             Prob > chi2      =  0.0000
```

sixmo_mortality	Odds ratio	Std. err.	z	P>|z|	[95% conf.	interval]
1.dilqts	1.098411	.0849174	1.21	0.225	.943972	1.278117
med						
CITALOPRAM	.7919387	.0463986	-3.98	0.000	.7060263	.8883053
DROPERIDOL	.7275804	.047939	-4.83	0.000	.6394357	.8278757
ESCITALOPRAM	.8011983	.0413039	-4.30	0.000	.7241997	.8863836
HALOPERIDOL	.8271307	.0257667	-6.09	0.000	.7781396	.8792062
HYDROXYCHLOROQUINE	.9297828	.0863143	-0.78	0.433	.7751078	1.115324
LEVOFLOXACIN	.9618982	.0355159	-1.05	0.293	.8947473	1.034089
METHADONE	.9112469	.0825171	-1.03	0.305	.7630557	1.088218
ONDANSETRON	.8136311	.0209758	-8.00	0.000	.7735407	.8557992
SOTALOL	.6570639	.0820085	-3.36	0.001	.5144803	.8391634
dilqts#med						
1#CITALOPRAM	.8525899	.14563	-0.93	0.350	.6100246	1.191607
1#DROPERIDOL	1.086542	.2344857	0.38	0.701	.711786	1.658607
1#ESCITALOPRAM	1.020535	.1558508	0.13	0.894	.7565485	1.376635
1#HALOPERIDOL	1.060405	.1018657	0.61	0.542	.8784207	1.28009
1#HYDROXYCHLOROQUINE	.6125018	.1741889	-1.72	0.085	.3507799	1.069498
1#LEVOFLOXACIN	.9875496	.1223667	-0.10	0.919	.774617	1.259015
1#METHADONE	.7509267	.1909163	-1.13	0.260	.4562336	1.23597
1#ONDANSETRON	.8479701	.0700364	-2.00	0.046	.7212358	.9969739
1#SOTALOL	.7637217	.194503	-1.06	0.290	.4636102	1.258106
age	1.037984	.0007967	48.57	0.000	1.036424	1.039547
female	.9066288	.0198712	-4.47	0.000	.8685067	.9464243
race_caucasian	.9412444	.0366003	-1.56	0.119	.8721747	1.015784
race_black	.871154	.0494366	-2.43	0.015	.7794542	.973642
ethnicity_hisp	.9104198	.0383267	-2.23	0.026	.8383164	.9887248
icu	1.938238	.0478866	26.79	0.000	1.846618	2.034403
tele	1.323003	.0300085	12.34	0.000	1.265476	1.383145
ob	.2609737	.1182257	-2.97	0.003	.1073959	.6341706
stepdown	1.234214	.0263727	9.85	0.000	1.183592	1.287001
cluster_4						
1	1.660222	.0790393	10.65	0.000	1.512316	1.822594
2	2.247144	.1183641	15.37	0.000	2.026728	2.491531
3	1.157537	.0547564	3.09	0.002	1.055041	1.26999
_cons	.0054052	.0004526	-62.34	0.000	.004587	.0063692

Note: **_cons** estimates baseline odds (conditional on zero random effects).

Supplemental Table 20. Multivariable marginal logistic regression model for impact of clinical decision-support (CDS) system on 1-year mortality in patients with drug-induced QT prolongation (diLQTS). Note that the P value for interaction of diLQTS across all medications (df = 9) was 0.0117 (chi-square 21.23), and for diLQTS independentaly P = 0.2712, df = 1, chi-square = 1.21.

```
GEE population-averaged model                 Number of obs    = 178,097
Group variable: arb_personid                  Number of groups = 102,847
Family: Binomial                              Obs per group:
Link:   Logit                                              min =       1
Correlation: exchangeable                                  avg =     1.7
                                                           max =      52
                                              Wald chi2(31)    = 6138.39
Scale parameter = 1                           Prob > chi2      =  0.0000
```

oneyr_mortality	Odds ratio	Std. err.	z	P>|z|	[95% conf.	interval]
1.dilqts	1.085796	.0668061	1.34	0.181	.9624455	1.224956
med						
CITALOPRAM	.8529682	.0368177	-3.68	0.000	.7837751	.9282698
DROPERIDOL	.9291998	.0410011	-1.66	0.096	.8522159	1.013138
ESCITALOPRAM	.8667733	.0330856	-3.75	0.000	.804293	.9341072
HALOPERIDOL	.8800303	.0206453	-5.45	0.000	.8404825	.921439
HYDROXYCHLOROQUINE	1.017436	.0700287	0.25	0.802	.8890377	1.164379
LEVOFLOXACIN	.9809237	.0273019	-0.69	0.489	.9288464	1.035921
METHADONE	1.006991	.0678672	0.10	0.918	.8823848	1.149193
ONDANSETRON	.8843855	.0171657	-6.33	0.000	.8513732	.9186779
SOTALOL	.707262	.0635634	-3.85	0.000	.5930355	.8434899
dilqts#med						
1#CITALOPRAM	.918302	.115876	-0.68	0.499	.7170948	1.175965
1#DROPERIDOL	.8793523	.1415086	-0.80	0.424	.6414819	1.205428
1#ESCITALOPRAM	1.003835	.1159616	0.03	0.974	.8004475	1.258902
1#HALOPERIDOL	1.020763	.0768086	0.27	0.785	.8807956	1.182972
1#HYDROXYCHLOROQUINE	.6544027	.1384305	-2.00	0.045	.4322992	.9906171
1#LEVOFLOXACIN	.906266	.0891729	-1.00	0.317	.7473102	1.099032
1#METHADONE	.7350316	.1440112	-1.57	0.116	.5006499	1.07914
1#ONDANSETRON	.852737	.0551251	-2.46	0.014	.7512582	.9679233
1#SOTALOL	.8935374	.1598107	-0.63	0.529	.6293234	1.268679
age	1.041681	.0007631	55.74	0.000	1.040187	1.043178
female	.8685492	.0180994	-6.76	0.000	.8337898	.9047577
race_caucasian	.9538413	.0357001	-1.26	0.207	.8863752	1.026443
race_black	.9286617	.0502052	-1.37	0.171	.8352951	1.032464
ethnicity_hisp	.9550695	.0383302	-1.15	0.252	.8828225	1.033229
icu	1.420798	.0281874	17.70	0.000	1.366612	1.477132
tele	1.28368	.0216979	14.77	0.000	1.24185	1.326919
ob	.3942322	.1254237	-2.93	0.003	.2113221	.7354602
stepdown	1.215474	.0211572	11.21	0.000	1.174706	1.257656
cluster_4						
1	1.331069	.047377	8.03	0.000	1.241377	1.427242
2	1.645237	.0678487	12.07	0.000	1.517488	1.78374
3	1.026888	.036031	0.76	0.450	.9586421	1.099992
_cons	.0065297	.0004806	-68.36	0.000	.0056525	.0075429

Note: **_cons** estimates baseline odds (conditional on zero random effects).
